# Bioluminescent reporter influenza A viruses to track viral infections

**DOI:** 10.1128/spectrum.02150-25

**Published:** 2025-10-08

**Authors:** Ramya S. Barre, Ahmed Mostafa, Kevin Chiem, Rebecca L. Pearl, Roy N. Platt, Anastasija Cupic, Timothy J. C. Anderson, Ulla G. Knaus, Randy A. Albrecht, Adolfo García-Sastre, James J. Kobie, Aitor Nogales, Luis Martinez-Sobrido

**Affiliations:** 1 Texas Biomedical Research Institute 7075, San Antonio, Texas, USA; 2 Department of Microbiology, Immunology, and Molecular Genetics, University of Texas Health Sciences Center at San Antonio 557944, San Antonio, Texas, USA; 3 Center of Scientific Excellence for Influenza Viruses, National Research Centre 68787, Giza, Egypt; 4 Department of Microbiology, Icahn School of Medicine at Mount Sinai 200769, New York, New York, USA; 5 Global Health Emerging Pathogens Institute, Icahn School of Medicine at Mount Sinai 5925, New York, New York, USA; 6 Graduate School of Biomedical Sciences, Icahn School of Medicine at Mount Sinai 5925, New York, New York, USA; 7 Conway Institute, School of Medicine, University College Dublin 37438, Dublin, Ireland; 8 Department of Medicine, Division of Infectious Diseases, Icahn School of Medicine at Mount Sinai 377569, New York, New York, USA; 9 Tisch Cancer Institute, Icahn School of Medicine at Mount Sinai 145753, New York, New York, USA; 10 Department of Pathology, Molecular and Cell-Based Medicine, Icahn School of Medicine at Mount Sinai 214369, New York, New York, USA; 11 Icahn Genomics Institute, Icahn School of Medicine at Mount Sinai 5925, New York, New York, USA; 12 Heersink School of Medicine, Infectious Diseases, University of Alabama at Birmingham 9967, Birmingham, Alabama, USA; 13 Center for Animal Health Research, CISA-INIA-CSIC 54459 , Madrid, Spain; Cornell University College of Veterinary Medicine, Ithaca, New York, USA

**Keywords:** seasonal influenza, bioluminescent reporter, nanoluciferase, *in vivo *imaging systems, H1N1, non-structural segment

## Abstract

Influenza A viruses (IAVs) infect a wide range of mammalian and bird species and are responsible for seasonal outbreaks and occasional pandemics of great consequences in humans. Studying IAVs requires methods to detect the presence of the virus in infected cells or animal models. Recombinant IAV-expressing fluorescent proteins has allowed monitoring of viral infection in cultured cells and *ex vivo* in the organs of infected animals. However, fluorescent-expressing IAVs are often attenuated and are not suited for the imaging of infected animals using *in vivo* imaging systems (IVISs). To overcome this limitation, we generated a recombinant pandemic influenza A/California/04/2009 H1N1 (pH1N1)-expressing nanoluciferase (Nluc) from the non-structural viral segment, hereafter referred to as pH1N1-Nluc. The pH1N1-Nluc replicates efficiently *in vitro*, with growth kinetics and plaque morphology comparable to wild-type pH1N1 (pH1N1-WT). We used this pH1N1-Nluc to demonstrate its ability to effectively identify neutralizing monoclonal antibodies and antivirals, with neutralization and inhibition results comparable to pH1N1-WT. In mice, pH1N1-Nluc was able to induce similar body weight loss and mortality, and viral titers comparable to pH1N1-WT, results that were also recapitulated in a ferret model of IAV infection. Using IVIS, pH1N1-Nluc enabled non-invasive, real-time tracking of viral infection *in vivo* and *ex vivo* following infection of mice with viral titers in tissues comparable to pH1N1-WT. The flexibility of this approach was further demonstrated by the generation of a Nluc-expressing recombinant A/Puerto Rico/8/1934 H1N1 (PR8-Nluc). Altogether, our results demonstrate that Nluc-expressing recombinant IAVs represent a valuable tool for *in vitro* and *in vivo* studies, including the identification of antivirals and/or neutralizing antibodies, and to assess the protective efficacy of vaccines.

**IMPORTANCE:**

Influenza A viruses (IAVs) pose a threat to human and animal health. Mechanisms that control IAV replication and pathogenesis are incompletely understood due to the lack of experimental approaches to visualize and quantify viral dynamics in real time. The use of replication-competent fluorescent-expressing IAV *in vivo* has been challenging because such viruses typically have reduced replication fitness and are not suited for imaging of entire animals. Herein, we developed replication-competent recombinant IAV-expressing nanoluciferase (Nluc) that can be used to visualize viral infection in living animals. Infection with Nluc-expressing IAV could be monitored in real time using *in vivo* imaging systems. Importantly, the Nluc reporter overcomes several shortcomings of fluorescent proteins and provides a new and sensitive tool to interrogate viral dynamics and immune responses *in vitro* and *in vivo*. This technology can be applied to advance studies and accelerate the development of new prophylactics and therapeutics against IAV.

## INTRODUCTION

Influenza viruses are respiratory pathogens of the family *Orthomyxoviridae*. Influenza viruses are classified into four different types: influenza A virus (IAV), influenza B virus (IBV), influenza C virus, and influenza D virus (1). IAV and IBV are currently circulating in humans and are responsible for seasonal infections that are associated with significant public health concerns and economic losses (1). IAV has also been responsible for causing pandemics of significant consequences to humans (2).

IAV is an enveloped virus with eight single-stranded negative-sense viral segments, namely polymerase basic 2 (PB2), polymerase basic 1 (PB1), polymerase acidic (PA), hemagglutinin (HA), nucleoprotein (NP), neuraminidase (NA), matrix protein (M), and non-structural protein (NS) segments (1), each encoding one or more proteins (1). The smallest segment, NS, encodes at least two viral proteins: the NS1 protein and the nuclear export protein (NEP), via mRNA splicing (1). The NS1 is a NS viral protein that plays a major role in virus replication and pathogenesis as an interferon (IFN)-antagonistic protein. NEP is involved in the export of viral RNAs from the host cell nucleus to the cytoplasm during viral infection (3).

In April 2009, a novel H1N1 IAV appeared in Mexico and the United States (4). This virus quickly spread worldwide, leading the World Health Organization (https://www.who.int/emergencies/situations/influenza-a-(h1n1)-outbreak) to declare it as the first influenza pandemic of the 21st century in June 2009 and the third influenza pandemic involving an IAV H1N1 subtype (1, 5). Since then, this pandemic H1N1 (pH1N1) IAV has become seasonal in the human population. This event highlights the importance of H1N1 IAV infections in human health and the recurring potential of IAV H1N1 to cause pandemics in humans.

To easily track IAV infections *in vitro* and *in vivo*/*ex vivo* in animal models, fluorescent or bioluminescent viruses were developed by modifying viral segments to introduce one or more reporter gene(s) (6–10). We and others have previously shown how IAV-expressing fluorescent and/or bioluminescent reporter genes from different viral segments, including PB2, PB1, PA, HA, NA, and NS, represent a suitable option to track viral infection in cultured cells and validated animal models of infection (3, 6–8, 11–19). In addition, we have also generated a bireporter IAV expressing a fluorescent reporter gene (Venus) from the NS segment and a bioluminescent nanoluciferase (Nluc) gene from the HA segment (9). One of the advantages of using recombinant virus-expressing fluorescent reporter genes is their ability to identify the presence of infected cells in cultured cells or *ex vivo,* from tissues of infected animals using *in vivo* imaging systems (IVISs) (9). However, recombinant IAV-expressing fluorescent proteins are attenuated both *in vitro* and *in vivo* (3, 10, 20, 21). Although recombinant IAV-expressing fluorescent reporter genes are suitable for *in vivo* imaging using IVIS, their utility is limited by the size or body mass of the infected animal and is frequently disturbed by tissue autofluorescence, resulting in substantial background (22, 23). Moreover, it has been shown that recombinant IAV-expressing fluorescent proteins could easily lose reporter expression after a few passages of the virus in cultured cells (10, 20, 22, 24). Bioluminescence (e.g., Nluc) offers the advantage of not affecting viral replication in cultured cells or in animal models and the feasibility of tracking viral infections in entire animals using IVIS (25). Thus, recombinant IAVs-expressing Nluc have the advantage over fluorescent recombinant viruses to track viral infections in the entire animal using IVIS (9, 11, 26). Several recombinant IAVs-expressing Nluc from the viral polymerases have been previously described in the literature. However, to date, few recombinant IAVs-expressing Nluc from the viral NS segment have been described for avian H9N2 (18), PR8 (19), or PR8-based H5N1 and PR8-based H7N9 (27). Expression of Nluc from the NS segment offers several advantages. These include minimal impact on viral fitness, stable reporter expression over multiple passages, and compatibility with sensitive, non-destructive luminescence-based assays (19). Notably, none of these studies have been conducted in a pandemic H1N1 background.

In this study, we generated a recombinant influenza A/California/04/2009 H1N1 (pH1N1)-expressing Nluc (pH1N1-Nluc) by introducing the Nluc open reading frame (ORF) fused to the C-terminal domain of the NS1 protein in a modified NS viral segment. *In vitro*, pH1N1-Nluc replicates and generates plaques of comparable size to pH1N1-WT. Importantly, we demonstrated that pH1N1-Nluc can be used to easily identify neutralizing monoclonal antibodies (MAbs) or antivirals with neutralization and inhibition titers, respectively, similar to those obtained with pH1N1-WT, opening the feasibility of using pH1N1-Nluc to interrogate large libraries of compounds in high-throughput screening (HTS) settings to identify neutralizing MAbs and/or antivirals. *In vivo,* pH1N1-Nluc has similar morbidity and mortality to pH1N1-WT but offers the opportunity to identify the presence of the virus in infected mice using IVIS. Notably, viral titers of pH1N1-Nluc in the nasal turbinate (NT) and lungs of infected animals were comparable to those in pH1N1-WT-infected mice, demonstrating the feasibility of using pH1N1-Nluc to evaluate viral pathogenicity, replication, and tissue tropism. We also evaluated viral infection and transmission of pH1N1-Nluc in ferrets and the feasibility of detecting the presence of the virus in tissues by Nluc signal. Importantly, pH1N1-Nluc shows genetic and phenotypic stability after serial passages in Madin-Darby canine kidney (MDCK) cells. The flexibility of this approach was further demonstrated by the generation of a Nluc-expressing recombinant influenza A/Puerto Rico/8/1934 H1N1 (PR8-Nluc). Altogether, our results demonstrate that Nluc-expressing recombinant IAVs represent a valuable tool for *in vitro* and *in vivo* studies, including the identification of neutralizing MAbs and/or antivirals, and to assess the protective efficacy of vaccines.

## RESULTS

### Generation and characterization of recombinant pH1N1-Nluc

**Fig 1:**
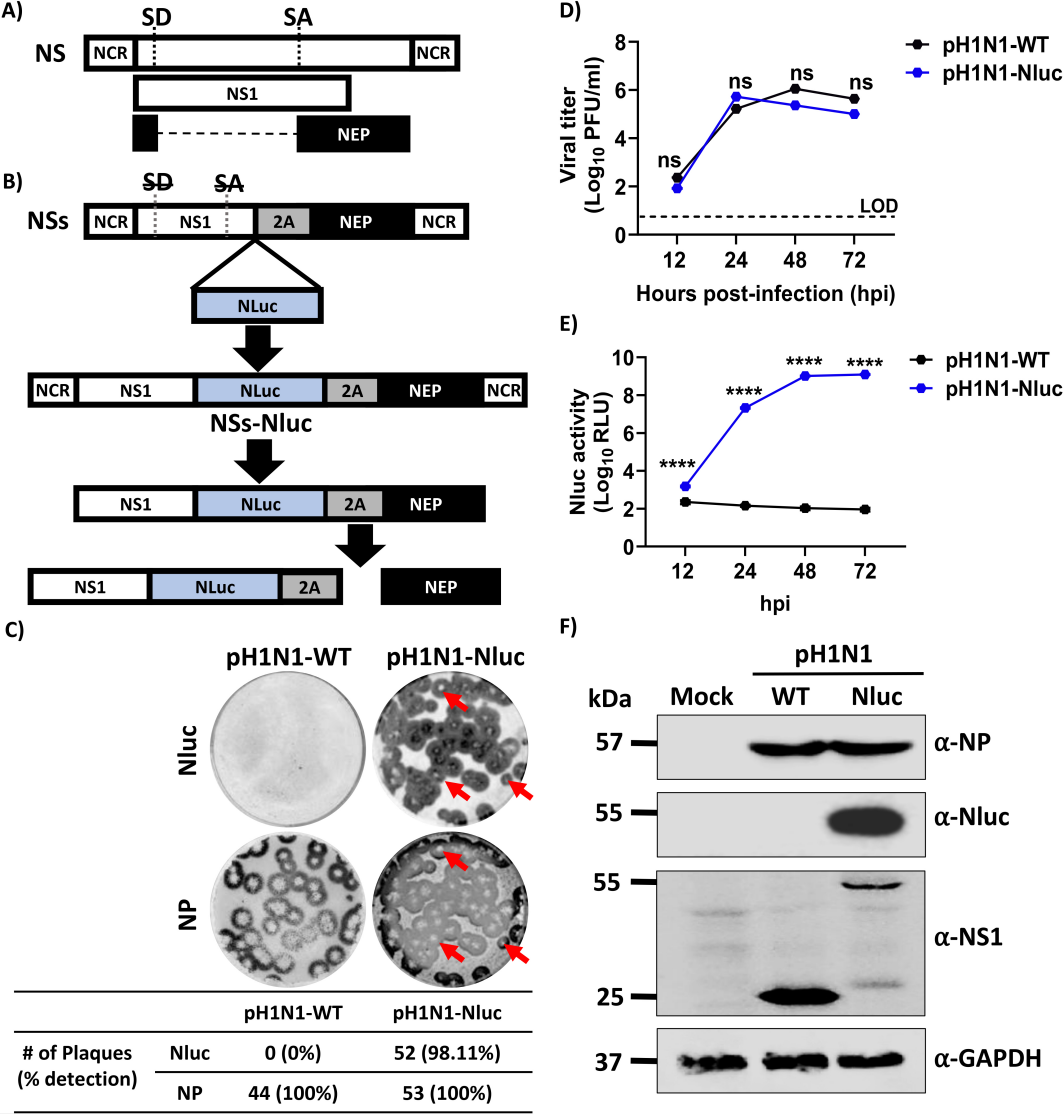
Generation and *in vitro* characterization of pH1N1-Nluc. (**A**) Schematic representation of the NS viral segment. The viral NS1 is represented in white and NEP in black. NCR, non-coding regions. (**B**) Schematic representation of NSs-Nluc viral segment encoding Nluc. The Nluc coding sequence is represented in blue and the PTV-1 2A in a gray box. To inhibit the splicing process in the NSs constructs, silent mutations were introduced in the splice donor (SD, strikethrough) and acceptor (SA, strikethrough) sites. (**C**) Plaque phenotype of pH1N1-WT and pH1N1-Nluc in MDCK cells. Viral plaques were evaluated at 72 hpi. Nluc staining (top) and NP immunostaining (bottom). Red arrows show the co-localization of Nluc staining (top) and viral plaques (bottom). (**D**) Multicycle growth kinetics of pH1N1-WT and pH1N1-Nluc viruses in MDCK cells. Viral titers from culture supernatants of pH1N1-WT and pH1N1-Nluc-infected (MOI 0.01) MDCK cells were determined using immunofocus assay at the indicated times post-infection. Data represent means and SD for triplicates. The dotted line indicates the limit of detection (LOD) of the assay. (**E**) Nluc expression in culture supernatants from MDCK cells infected with pH1N1-WT- and pH1N1-Nluc. Cell culture supernatants from the viral growth kinetics (**D**) were used to measure Nluc activity. (**F**) Western blots. MDCK cells were infected (MOI = 3) with pH1N1-WT or pH1N1-Nluc, or mock-infected. At 12 hpi, cell extracts were prepared, and a Western blot was performed to assess levels of NP, NS1, and Nluc expression. Cellular glyceraldehyde-3-phosphate dehydrogenase (GAPDH) was used as a loading control. Data are represented as mean ± SD. A two-way repeated measures ANOVA with Geisser-Greenhouse correction. Post hoc multiple comparisons were performed using Šídák to compare groups within each time point. The significant differences are indicated (ns = non-significant, ****P  <  0.0001).

To generate a replication-competent recombinant pH1N1-expressing Nluc, the Nluc ORF without the stop codon was cloned into the NS segment (Fig. 1) as previously described (7, 26). The IAV NS segment encodes NS1 and NEP using an alternative splicing mechanism (Fig. 1A). We first constructed a modified NS split (NSs) segment, where NS1 and NEP are expressed from a single transcript using the porcine teschovirus-1 (PTV-1) 2A autoproteolytic cleavage, resulting in the expression of a NS1-2A-NEP transcript that is independently translated into NS1 and NEP (Fig. 1B). Then, the Nluc ORF was cloned at the C-terminal of NS1 to generate the NSs plasmid-encoding Nluc (NSs-Nluc) to produce the recombinant pH1N1-Nluc virus (Fig. 1B). We next used our previously described plasmid-based reverse genetics approaches, where the pH1N1 NS plasmid was substituted with the NSs-Nluc plasmid together with the other seven plasmids encoding the rest of the pH1N1 viral segments to generate the pH1N1-Nluc virus.

After viral rescue, we next compared the plaque and replication phenotypes of pH1N1-Nluc to the wild-type pH1N1 (pH1N1-WT) (Fig. 1C and D). Interestingly, the plaque phenotype of pH1N1-Nluc was comparable to pH1N1-WT (Fig. 1C). Importantly, when viral plaques were stained with the anti-NP MAb HB-65, 98.11% of the viral plaques were Nluc positive by immunostaining (Fig. 1C). When we evaluated viral growth kinetics, pH1N1-Nluc replicated to levels comparable to pH1N1-WT in MDCK cells at defined time points post-infection (Fig. 1D). As expected, we were able to detect Nluc expression in the cell culture supernatants of MDCK cells infected with pH1N1-Nluc but not in cell culture supernatants of MDCK cells infected with pH1N1-WT (Fig. 1E). We further demonstrated Nluc expression from cell extracts obtained from MDCK cells infected with pH1N1-Nluc by Western blot (Fig. 1F). As expected and due to the carboxy-terminal extension with Nluc of the NS1 protein, we observed a higher molecular size of NS1 in MDCK cell extracts infected with pH1N1-Nluc compared to cells infected with pH1N1-WT (Fig. 1F). Notably, levels of expression of viral NP were comparable in MDCK cells infected with pH1N1-Nluc or pH1N1-WT (Fig. 1F). Although NS1 protein levels detected by Western blot were not apparently equivalent, the subcellular distribution of NS1 (nuclear and cytoplasmic localization) was similar in MDCK cells infected with pH1N1-WT or pH1N1-Nluc at 8 hours post-infection (hpi) (Fig. S1A). These results demonstrate that the recombinant pH1N1-Nluc has plaque and replication phenotypes that are similar to pH1N1-WT, with the advantage of easily monitoring viral infection by assessing Nluc expression from the cell culture supernatants of infected MDCK cells.

### pH1N1-Nluc efficiently inhibits IFNβ promoter activation

**Fig 2:**
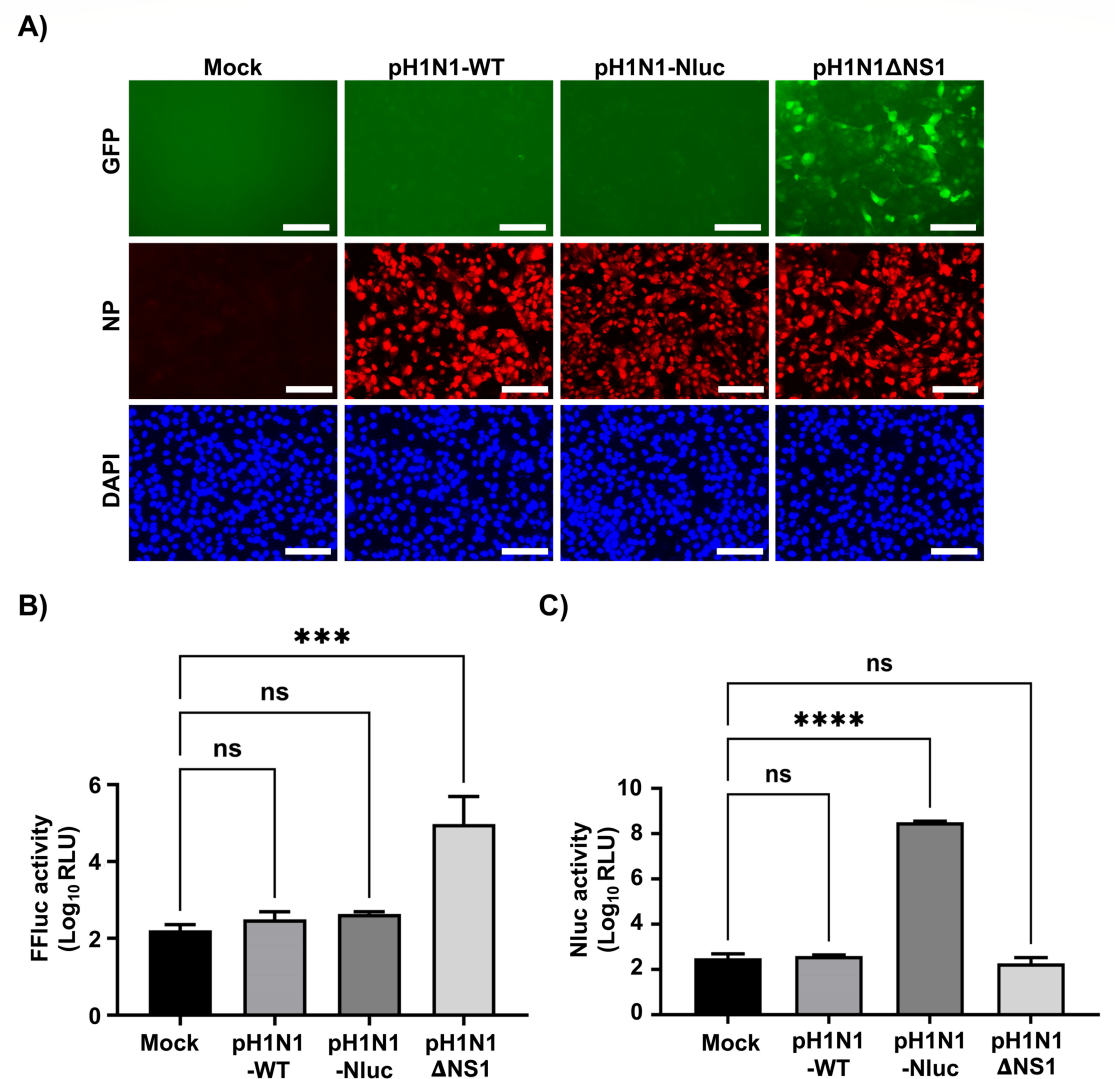
pH1N1-Nluc inhibits IFNβ promoter activation. MDCK IFNβ-GFP/IFNβ-FFluc cells were mock-infected or infected (MOI = 1) with pH1N1-WT, pH1N1-Nluc, or pH1N1 ΔNS1. At 12 hpi, IFNβ promoter activation was analyzed by GFP (**A**) and FFluc (**B**) expression. Nluc expression in the cell culture supernatants of infected cells was evaluated at the same time after viral infection (**C**). Scale bar: 100 µm. Data are represented as mean ± SD. A Welch’s one-way ANOVA was performed. *Post hoc* multiple comparisons were performed using the Dunnett method to compare groups within each time point. The significant differences are indicated (ns = non-significant, ****P  <  0.0001 and ***P  <  0.001).

Influenza NS1 protein functions as a key antagonist of the host antiviral response by inhibiting IFN signaling during viral infection (28). To assess whether modification of the NS segment in NSs or fusion of Nluc to the C-terminus of NS1 in pH1N1-Nluc (Fig. 1B) alters the ability of NS1 to suppress IFN responses, we utilized MDCK IFNβ-green fluorescent protein (GFP)/IFNβ-FFluc reporter cells (29). These cells express GFP and firefly luciferase (FFluc) under the control of the IFNβ promoter (29). Cells were infected at a multiplicity of infection (MOI) of 1 with pH1N1-WT or pH1N1-Nluc. As a positive control for IFN induction, parallel infections were performed with an NS1-deficient virus (pH1N1 ΔNS1), whereas mock-infected cells served as a negative control. At 12 hpi, IFNβ promoter activation was evaluated by monitoring GFP expression via fluorescence microscopy (Fig. 2A) and quantifying FFluc activity (Fig. 2B) using a luciferase plate reader. Robust GFP and FFluc expression was detected in cells infected with pH1N1 ΔNS1 (Fig. 2A and B). In contrast, no activation of the IFNβ promoter was observed in cells infected with pH1N1-WT or pH1N1-Nluc by fluorescent (Fig. 2A) or luciferase (Fig. 2B) activities, despite comparable levels of viral infection confirmed by NP immunostaining (Fig. 2A). These findings demonstrate that pH1N1-Nluc retains the ability to efficiently suppress IFNβ promoter activation upon viral infection, similar to the parental pH1N1-WT (Fig. 2C). As expected, Nluc expression was detected exclusively in cell culture supernatants from MDCK IFNβ-GFP/IFNβ-FFluc cells infected with pH1N1-Nluc (Fig. 2C), confirming functional reporter expression.

### Application of pH1N1-Nluc for the identification of MAbs and antivirals

**Fig 3:**
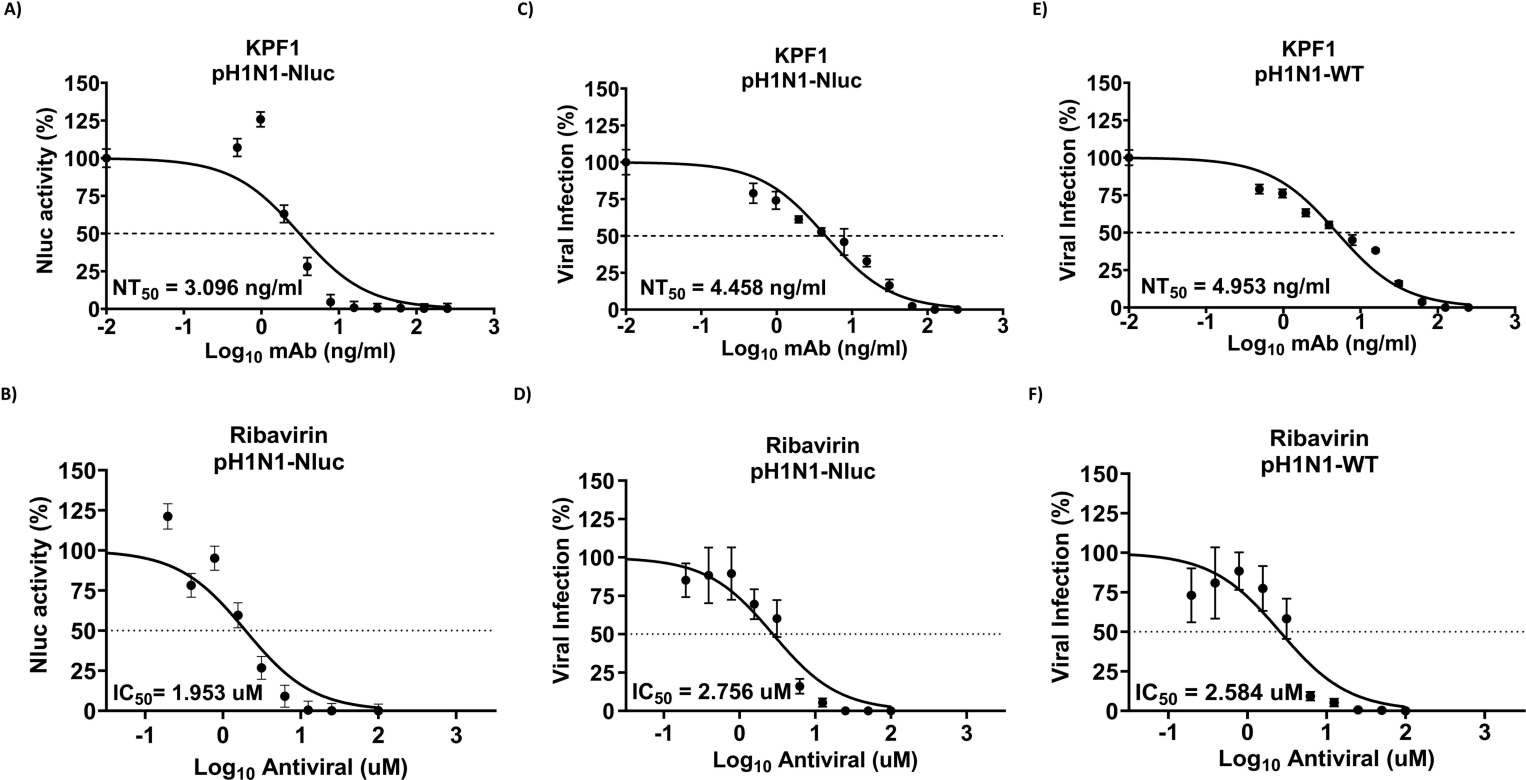
Microneutralization and antiviral assays to evaluate neutralizing antibodies and antivirals. Monolayers of MDCK cells (96-well plate format, 10E4 cells/well, quadruplicates) were infected with 100 plaque-forming units (PFU) of pH1N1-Nluc or pH1N1-WT virus for 1 h. After viral absorption, the virus inocula were removed, and cells were incubated with twofold serial dilutions of a KPF1 pH1N1 neutralizing antibody (starting concentration of 0.25 µg/mL) or Ribavirin (starting concentration of 100 µM). Virus neutralization and inhibition of pH1N1-Nluc were quantified by Nluc expression (**A and B**) using a luminometer or immunostaining titration of the viral titers (**C and D**) at 45 hpi. Immunostaining was used to determine the viral titers in supernatants from pH1N1-WT-infected and treated samples (**E and F**). The NT_50_ of KPF1 and the IC_50_ of Ribavirin were calculated using a sigmoidal dose-response curve. The dotted line indicates 50% neutralization or inhibition.

Neutralizing MAbs and antiviral therapeutics are important in protecting against (30–32). Most IAV neutralizing and/or antiviral assays rely on detecting the presence of infected cells using secondary biochemical methods, which typically extend the duration of the assay (15, 33). To overcome this limitation, we evaluated the feasibility of using Nluc expression to identify neutralizing MAbs and antivirals against pH1N1 (Fig. 3). To that end, we used a previously described neutralizing MAb (KPF1) (34, 35) and Ribavirin, a nucleoside analog previously shown to have antiviral activity against RNA and DNA viruses, including IAV (36–38). Importantly, we compared the neutralization and antiviral results using the Nluc-based microneutralization/antiviral assays to previously described assays (34) and calculated the neutralization titer 50 (NT_50_) and the inhibitory concentration 50 (IC_50_) of KPF1 MAb and ribavirin, respectively. Moreover, we compared the results obtained with pH1N1-Nluc to those obtained with pH1N1-WT. The NT_50_ of KPF1 using Nluc activity (NT_50_ = 3.096 ng/mL) (Fig. 3A) was comparable to the neutralization activity of KPF1 using a conventional microneutralization assay based on percentage of infection achieved by pH1N1-Nluc (NT_50_ = 4.458 ng/mL) (Fig. 3C) or by pH1N1-WT (NT_50_ = 4.953 ng/mL) (Fig. 3E). Likewise, the IC_50_ of ribavirin using the pH1N1-Nluc in the Nluc-based antiviral assay (IC_50_ = 1.953 ng/mL) (Fig. 3B) was comparable to that obtained with pH1N1-Nluc (Fig. 3D) or pH1N1-WT (Fig. 3F) using the classical antiviral assay (IC_50_ = 2.576 ng/mL and IC_50_ = 2.584 ng/mL, respectively). These results demonstrate the feasibility of using pH1N1-Nluc in Nluc-based assays to identify neutralizing MAbs or compounds with antiviral activity against pH1N1-WT with similar NT_50_ and IC_50_ values.

### pH1N1-Nluc retains the virulence of pH1N1-WT in C57BL/6J mice

**Fig 4:**
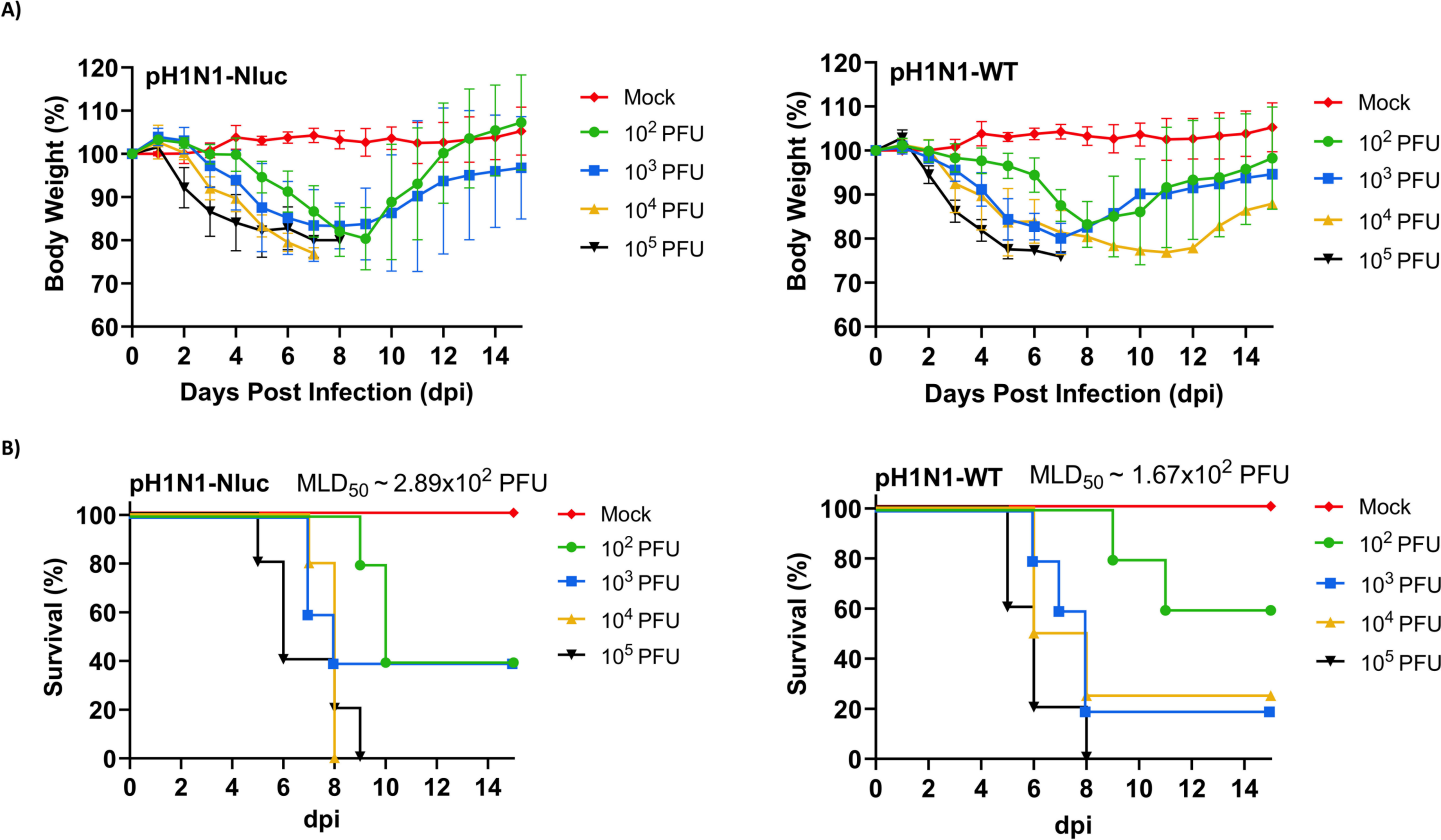
Pathogenicity of pH1N1-Nluc in mice. Female 6-week-old C57BL/6J mice (*n* = 5) were inoculated with 10^2^, 10^3^, 10^4^, and 10^5^ PFU of pH1N1-Nluc or pH1N1-WT and monitored daily for 14 days. Mock-infected mice were used as controls. (**A**) Percentage of body weight changes in mock-, pH1N1-Nluc-, and pH1N1-WT-infected C57BL/6J mice. (**B**) Survival rates of mock-, pH1N1-Nluc-, and pH1N1-WT-infected C57BL/6J mice. Mice that lost 25% or greater of their initial weight were sacrificed. The mock-infected group (*n* = 4) was the same for both pH1N1-Nluc- and pH1N1-WT-infected groups. The MLD_50_ was calculated by the Reed and Muench method. Data represent the means ± SD of the results for individual mice.

We next evaluated and compared the virulence of pH1N1-Nluc and pH1N1-WT in C57BL/6J (B6) mice (Fig. 4). To evaluate morbidity and mortality, B6 mice (*n* = 5) were infected, intranasally, with different doses (10^2^–10^5^ plaque-forming units [PFU]) of pH1N1-Nluc (Fig. 4A) or pH1N1-WT (Fig. 4B) and mice were monitored for changes in body weight and survival. A mock-infected group was also included as a control. To reduce the number of mice used in this study, the mock-infected group was the same for the pH1N1-Nluc and pH1N1-WT-infected groups. All mice infected with 10⁵ PFU of pH1N1-Nluc or pH1N1-WT rapidly lose weight and succumb to viral infection (Fig. 4A and B). All B6 mice infected with 10⁴ PFU of pH1N1-Nluc also succumbed to infection, while one mouse survived in the group infected with the same 10⁴ PFU dose of pH1N1-WT, although this mouse presented signs of infection (Fig. 4A and B). At 10³ PFU, two out of five mice survived infection with pH1N1-Nluc, while only one mouse survived in the pH1N1-WT group (Fig. 4A and B). When infected with 10² PFU, two mice survived infection with pH1N1-Nluc, and three mice survived infection with pH1N1-WT (Fig. 4A and B). The median lethal dose 50 (MLD₅₀) values were ~2.89 × 10² PFU for pH1N1-Nluc and ~1.67 × 10² PFU for pH1N1-WT (Fig. 4B). These findings indicate that pH1N1-Nluc retains the virulence and lethality of pH1N1-WT in B6 mice.

**Fig 5:**
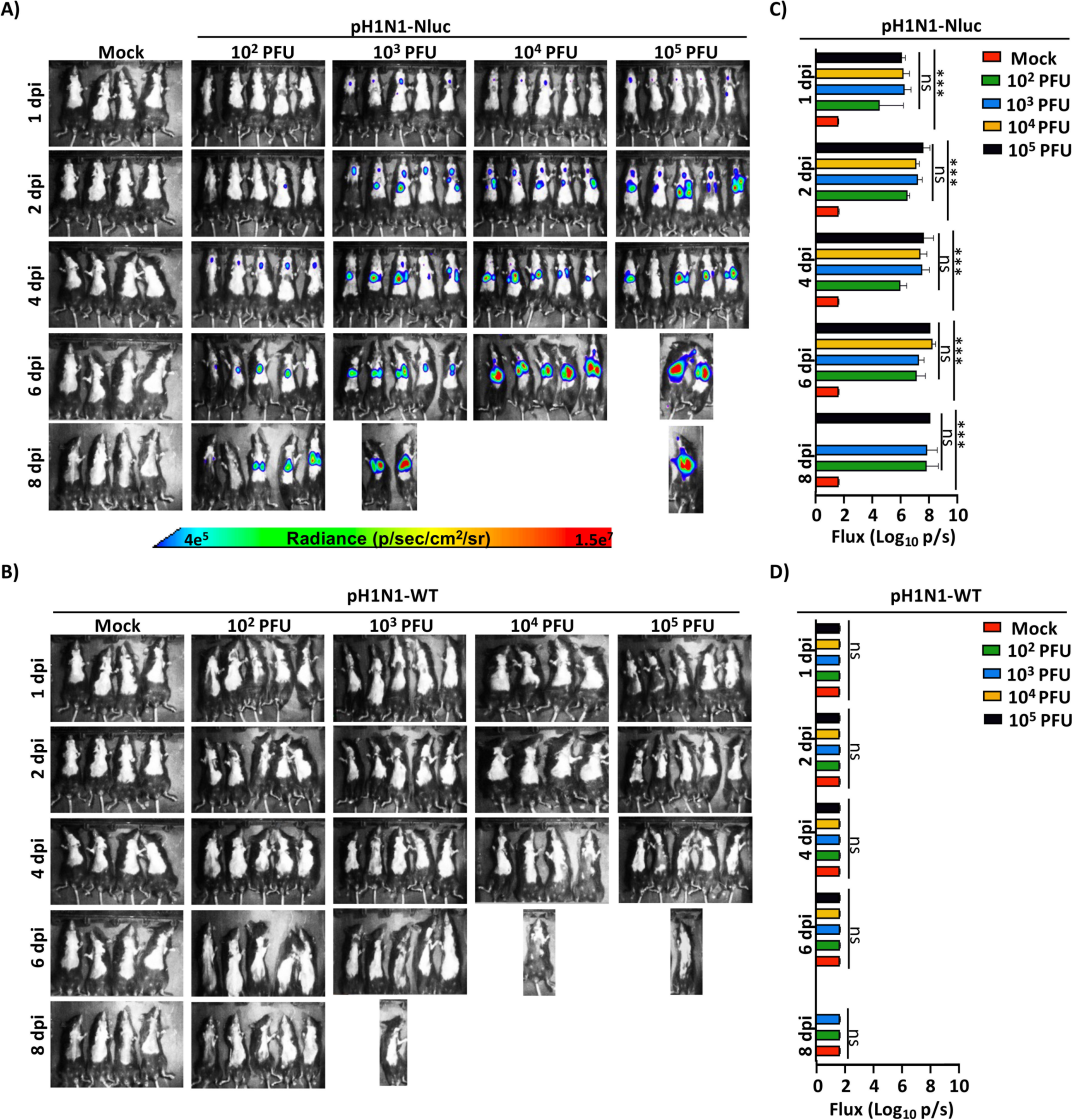
*In vivo* Nluc expression in pH1N1-Nluc-infected C57BL/6 mice. Female 6-week-old C57BL/6J mice infected as in Fig. 4 were monitored for Nluc luminescence at 1, 2, 4, 6, and 8 days post-infection (dpi) with pH1N1-Nluc (**A**) or pH1N1-WT (**B**) using IVIS. Mock-infected animals in the control group were the same for both pH1N1-Nluc- (**A**) and pH1N1-WT- (**B**) infected groups. Radiance, defined as the number of photons per s per square cm per steradian (p s−1 cm−2 sr−1), is shown on the heat maps at the bottom. Quantification of Nluc luminescence in C57BL/6 mice infected with pH1N1-Nluc (**C**) or pH1N1-WT (**D**). Data for 8 dpi are not shown due to the absence of surviving mice. A mixed-effects ANOVA followed by Tukey *post hoc* multiple comparisons test was used (ns = non-significant, ***P  <  0.0001).

One of the advantages of using pH1N1-Nluc is the ability to track viral infection in living mice using *IVIS*. Consequently, mock-infected and pH1N1-Nluc- or pH1N1-WT-infected B6 mice were imaged with IVIS at 1, 2, 4, 6, and 8 days post-infection (dpi) (Fig. 5). The mock-infected group was common for both pH1N1-Nluc- and pH1N1-WT-infected mice. We detected Nluc signal in mice infected with pH1N1-Nluc (Fig. 5A). Nluc level was both dose and time dependent as we were able to detect an increase in the Nluc signal from day 1 to day 8, and mice infected with the highest dose showed higher levels of Nluc expression (Fig. 5A). Notably, we were able to detect Nluc signals in the trachea of infected mice at early times post-infection (Fig. 5A) that progress to the lungs at later times during viral infection (Fig. 5A). Nluc level increased gradually but non-significantly from day 1 to day 8 in mice, with lowest levels of Nluc expression observed in mice infected with all doses of pH1N1-Nluc at day 1 post-infection (Fig. 5C). A dose- and time-dependent increase in Nluc luminescence was observed between 10^2^ PFU and 10^3–5^ PFU of pH1N1-Nluc, with no significant difference in luminescence beyond 10^3^ PFU, indicating a saturation effect at higher doses (Fig. 5A and C). As expected, we were not able to detect Nluc expression in B6 mice infected with pH1N1-WT (Fig. 5B and D).

**Fig 6:**
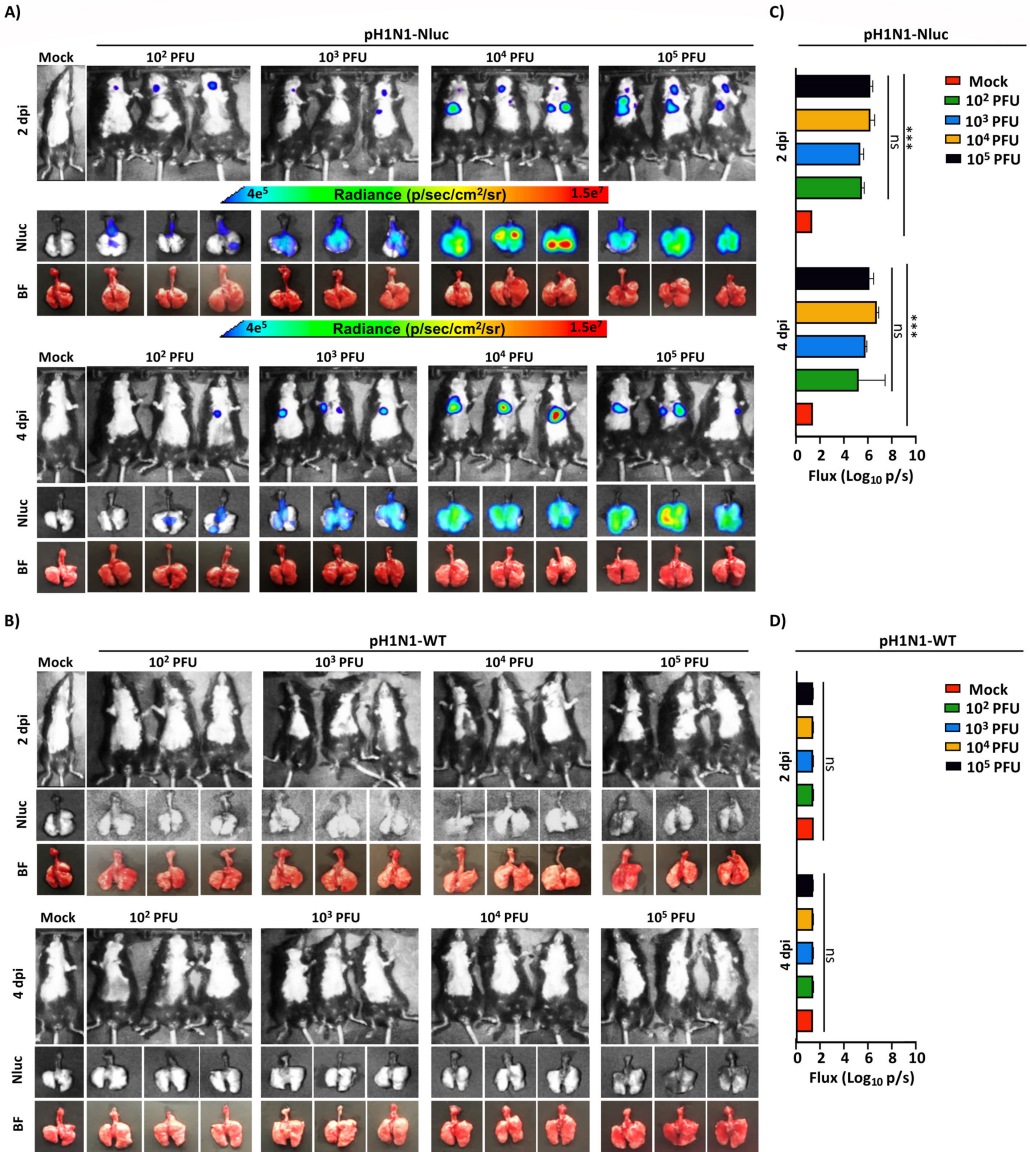
*In vivo* and *ex vivo* imaging of C57BL/6J mice infected with pH1N1-Nluc. (**A**) Female 6-week-old C57BL/6J mice were infected with the indicated doses of pH1N1-Nluc. At 2 and 4 dpi, Nluc luminescence was detected using IVIS. (**B**) Female 6-week-old C57BL/6J mice were infected with the indicated doses of pH1N1-WT, and luminescence was monitored using IVIS at 2 and 4 dpi. The mock-infected group was common for both the pH1N1-Nluc- (**A**) and the pH1N1-WT- (**B**) infected groups. Quantifications of Nluc luminescence in C57BL/6J mice infected with pH1N1-Nluc (**C**) or pH1N1-WT (**D**). Radiance, defined as the number of photons per s per square cm per steradian (p s−1 cm−2 sr−1), is shown on each of the indicated heat maps. A mixed-effects ANOVA followed by a Tukey *post hoc* multiple comparisons test was used (ns = non-significant, ***P  <  0.001).

We next assessed Nluc level *ex vivo*, in the lungs of animals infected with pH1N1-Nluc (Fig. 6). Briefly, different groups of mock-infected and pH1N1-Nluc- or pH1N1-WT- infected (10^2^–10^5^ PFU) B6 mice (*n* = 6/group) were monitored for Nluc luminescence using IVIS before surgically excising the NT and lungs on 2 and 4 dpi (*n* = 3/group/day) (Fig. 6A). Same mock-infected mice were used for both the pH1N1-Nluc- (Fig. 6A) and the pH1N1-WT- (Fig. 6B) infected groups. Necropsied lungs were imaged *ex vivo* in the IVIS to detect luminescence (Fig. 6A). Similar to results *in vivo* (Fig. 5), Nluc luminescence in pH1N1-Nluc-infected B6 mice was dose dependent (Fig. 6A and B), with higher levels of Nluc expression observed in the lungs of mice infected with higher viral doses (10^3–5^ PFU) (Fig. 6A and C). As expected, we were not able to detect Nluc expression in the excised lungs of mice infected with pH1N1-WT (Fig. 6B and D).

**Fig 7:**
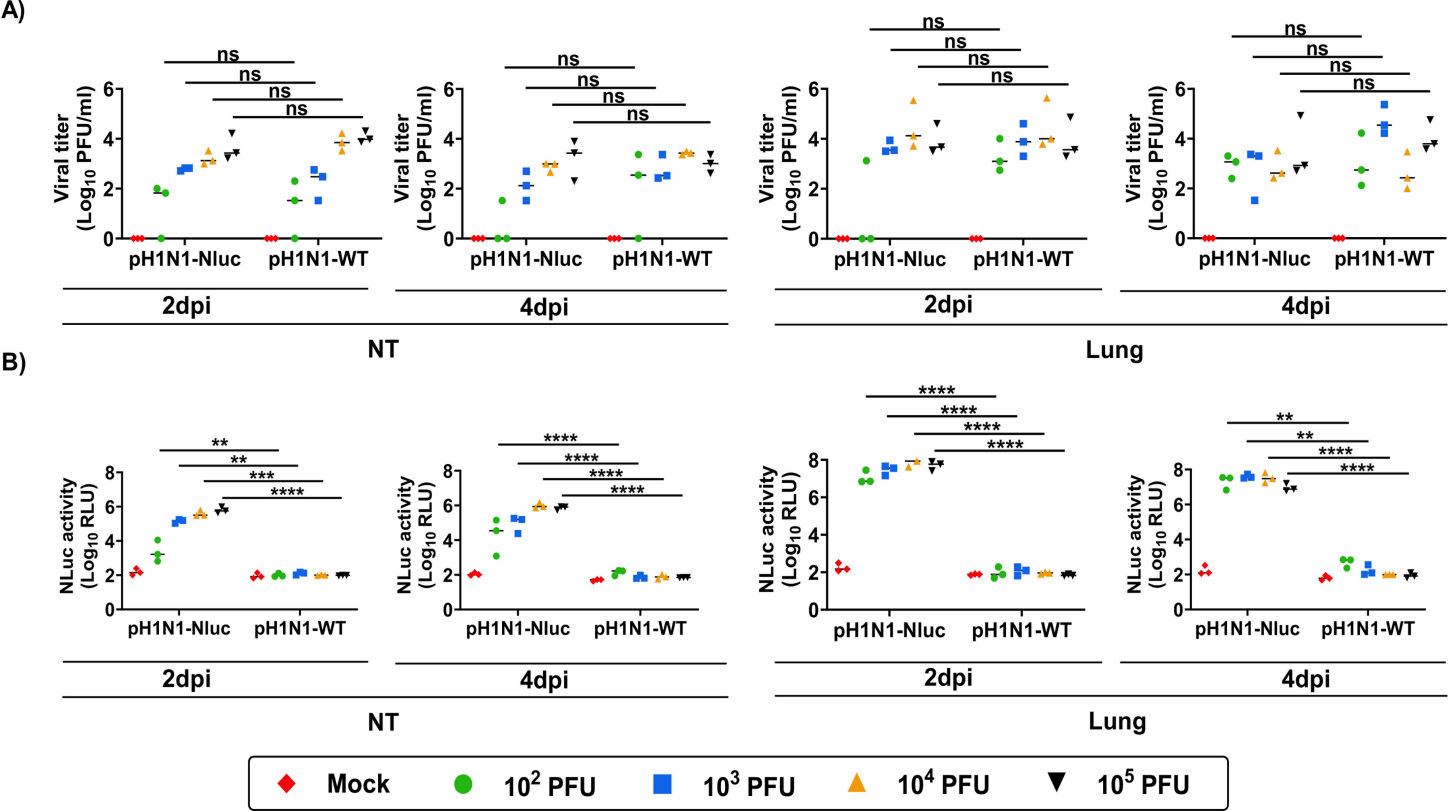
Viral titers and Nluc expression in C57BL/6J mice infected with pH1N1-Nluc. (**A**) Viral titers in the NT and lung tissues collected at 2 and 4 dpi are represented as Log_10_ PFU/mL. (**B**) Nluc expression in tissue homogenates collected from the same NT and lung tissues of mice infected with pH1N1-Nluc and pH1N1-WT. Data are represented as mean ± SD. A mixed-effects ANOVA followed by a Tukey *post hoc* multiple comparisons test was used (ns = non-significant, *P  <  0.05, **P  <  0.01, ***P  <  0.001, ****P  <  0.0001).

A dose-dependent increase in viral titers was observed in the NTs of mice infected with pH1N1-Nluc (Fig. 7A). Importantly, viral titers in the NT and lungs of B6 mice infected with pH1N1-Nluc were comparable to those detected in pH1N1-WT-infected B6 mice (Fig. 7A), with no significant differences. Notably, we were able to detect Nluc signals in the NT and lungs of B6 mice infected with pH1N1-Nluc (Fig. 7B). Nluc signals were dose-dependent as we observed higher levels of luminescence in mice infected with the highest dose of pH1N1-Nluc (Fig. 7B). We were not able to detect Nluc signals in the NT and lungs of B6 mice infected with pH1N1-WT (Fig. 7B). Altogether, these results indicate that pH1N1-Nluc replicated in the NT and lungs of infected B6 mice at levels comparable to pH1N1-WT and Nluc luminescence can be used as a valid surrogate to evaluate the presence of the virus in these tissues (Fig. 7A and B).

### Virulence and transmissibility of pH1N1-Nluc virus in ferrets

**Fig 8:**
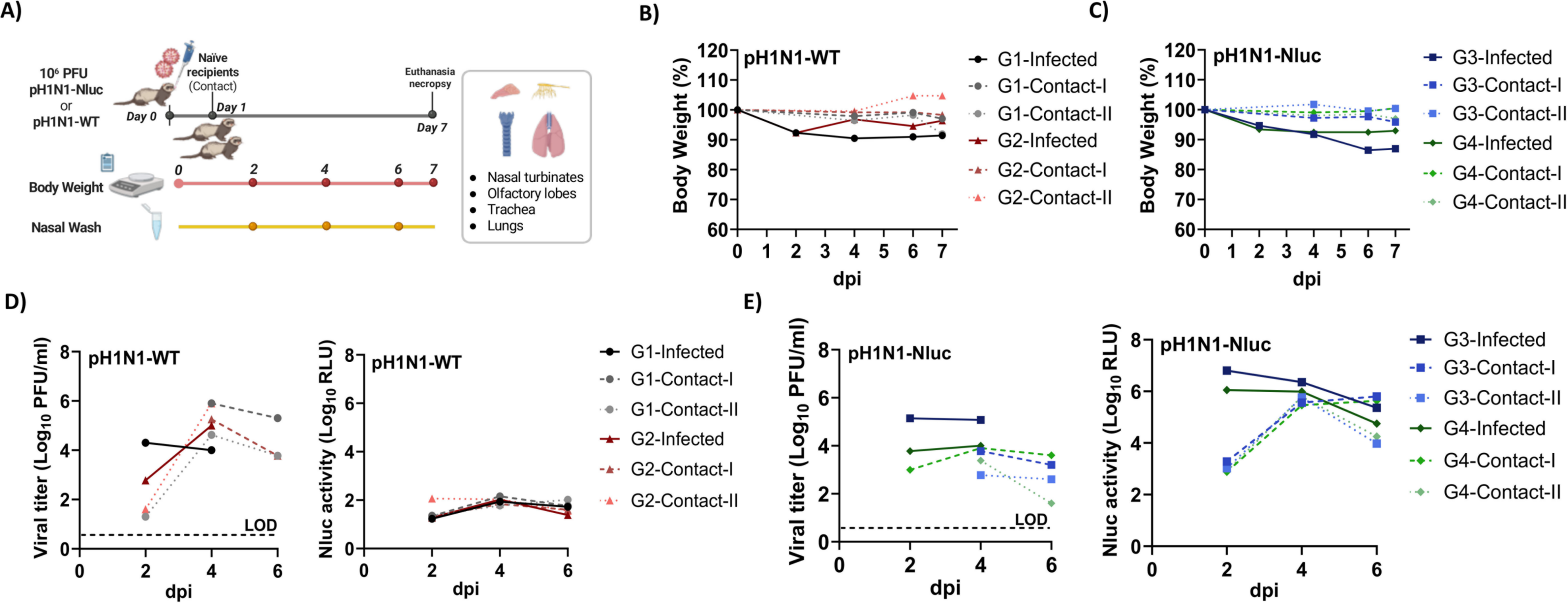
pH1N1-Nluc replication and transmission in ferrets. (**A**) Experimental design of the ferret experiment. (**B and C**) Percentages of body weight of ferrets infected (*n* = 2) with pH1N1-WT (**B**) and pH1N1-Nluc (**C**) and contact ferrets (*n* = 4). (**D and E**) Nasal washes from pH1N1-WT- and pH1N1-Nluc-infected ferrets and their direct contacts, respectively, were titrated by plaque assay (left) or directly assessed for Nluc activity (right). The dotted line indicates the limit of detection (LOD) of the assay.

Ferrets have become one of the most used mammalian animal models to study influenza virus infection and transmission due to their susceptibility to IAV infection and similarity to humans in respiratory physiology and disease progression (39–41). To investigate the impact of the presence of Nluc in pH1N1-Nluc virus on viral virulence and transmissibility in ferrets, we infected separately 2 ferrets with 10^6^ PFU of pH1N1-Nluc or pH1N1-WT via intranasal route and caged individually before adding two naïve ferrets to each cage as contacts at 24 hpi (Fig. 8A). Then, we measured body weight and collected nasal washes at 2, 4, and 6 dpi to determine viral titers in both infected and contact animals. Both pH1N1-WT (Fig. 8B) and pH1N1-Nluc (Fig. 8C) induced comparable levels of body weight loss (<15%) in experimentally infected ferrets, albeit no body weight loss in contact animals up to 7 dpi. In ferrets infected with pH1N1-WT (Fig. 8D) or pH1N1-Nluc (Fig. 8E), viral titers in nasal washes increased from 2 to 4 dpi and were undetectable by 6 dpi. In contact ferrets, viral titers also increased from 2 to 4 dpi and began to decline by 6 dpi (Fig. 8D and E). We detected Nluc luminescence in the nasal washes of pH1N1-Nluc-infected and contact ferrets, with Nluc levels decreasing from 2 to 6 dpi in infected ferrets, and Nluc levels increasing from 2 to 6 dpi in contact ferrets (Fig. 8E), which correlated with viral titers determined by plaque assay from the same nasal washes (Fig. 8E). By 6 dpi, virus clearance had occurred in directly infected (DI) ferrets with both pH1N1-WT and pH1N1-Nluc, resulting in undetectable virus shedding (Fig. 8E). The Nluc signal was not detected in the nasal washes of the pH1N1-WT-infected ferrets (Fig. 8D).

**Fig 9:**
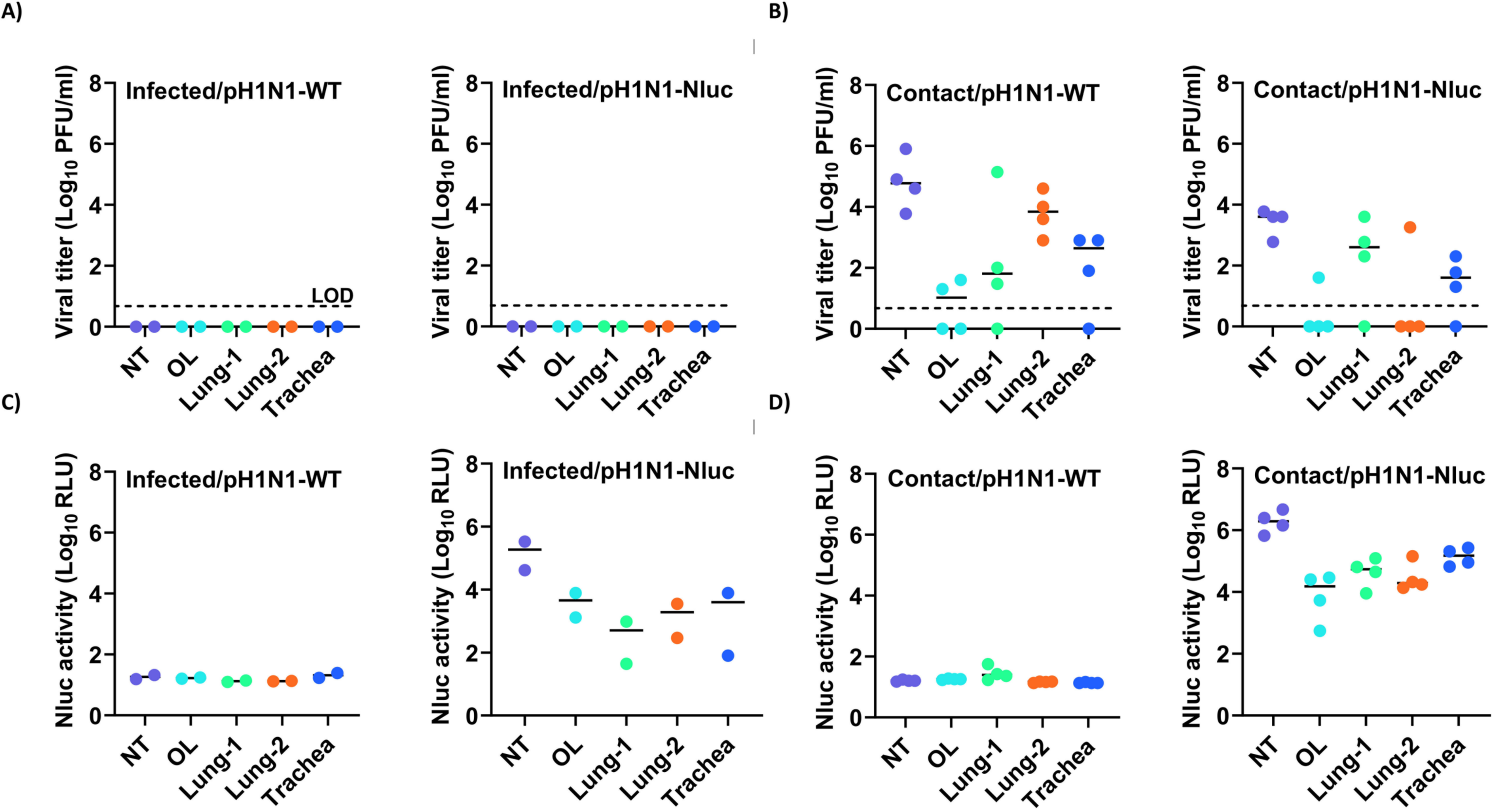
Viral titers and Nluc luminescence in pH1N1-Nluc- and pH1N1-WT-infected ferrets. (**A and B**) Viral titers in the NT, OL, lungs, and trachea infected (*n* = 2) with pH1N1-WT and pH1N1-Nluc (**A**), and direct contact (*n* = 4) animals (**B**), are represented as Log_10_ PFU/mL. (**C and D**) Nluc expressions in the NT, OL, lungs, and trachea of DI ferrets with pH1N1-Nluc and pH1N1-WT (**C**), and direct contact animals (**D**). The limit of detection (LOD) is indicated with a dashed line. Data are represented as mean ± SD.

To investigate virus tropism, the NT, olfactory lobe (OL), trachea, and lungs of infected and contact ferrets were collected at 7 dpi and homogenized to evaluate viral titers. At 7 dpi, no viral titers were detected in tissues from ferrets experimentally infected with pH1N1-WT and pH1N1-Nluc after recovery (Fig. 9A). However, we were able to detect high viral titers in the tissues collected from contact animals, with the highest levels in NT (Fig. 9B), indicating recent and active infection with the virus following contact transmission from the infected animals. Levels of pH1N1-Nluc in the different tissues in contact ferrets were slightly lower than those in pH1N1-WT contact ferrets (Fig. 9B). Importantly, no Nluc signal was observed in pH1N1-WT-infected or their contact ferrets (Fig. 9C and D). In contrast, we detect high levels of Nluc luminescence in tissues from both pH1N1-Nluc-infected and contact ferrets, with the highest levels of Nluc expression in NT (Fig. 9C and D). Nluc was detected in the tissues of experimentally infected animals on day 7 at a time when infectious viruses were not detectable, indicating either higher sensitivity of the Nluc assay or higher stability of Nluc in tissues as compared to infectious viruses (Fig. 9A and B, right panels). These results demonstrate that while pH1N1-Nluc virus was slightly attenuated compared to pH1N1-WT in ferrets, we were able to detect Nluc luminescence that correlates with viral infection in the tissues of both experimentally infected and contact animals.

### Genetic and phenotypic stability of pH1N1-Nluc

**Fig 10:**
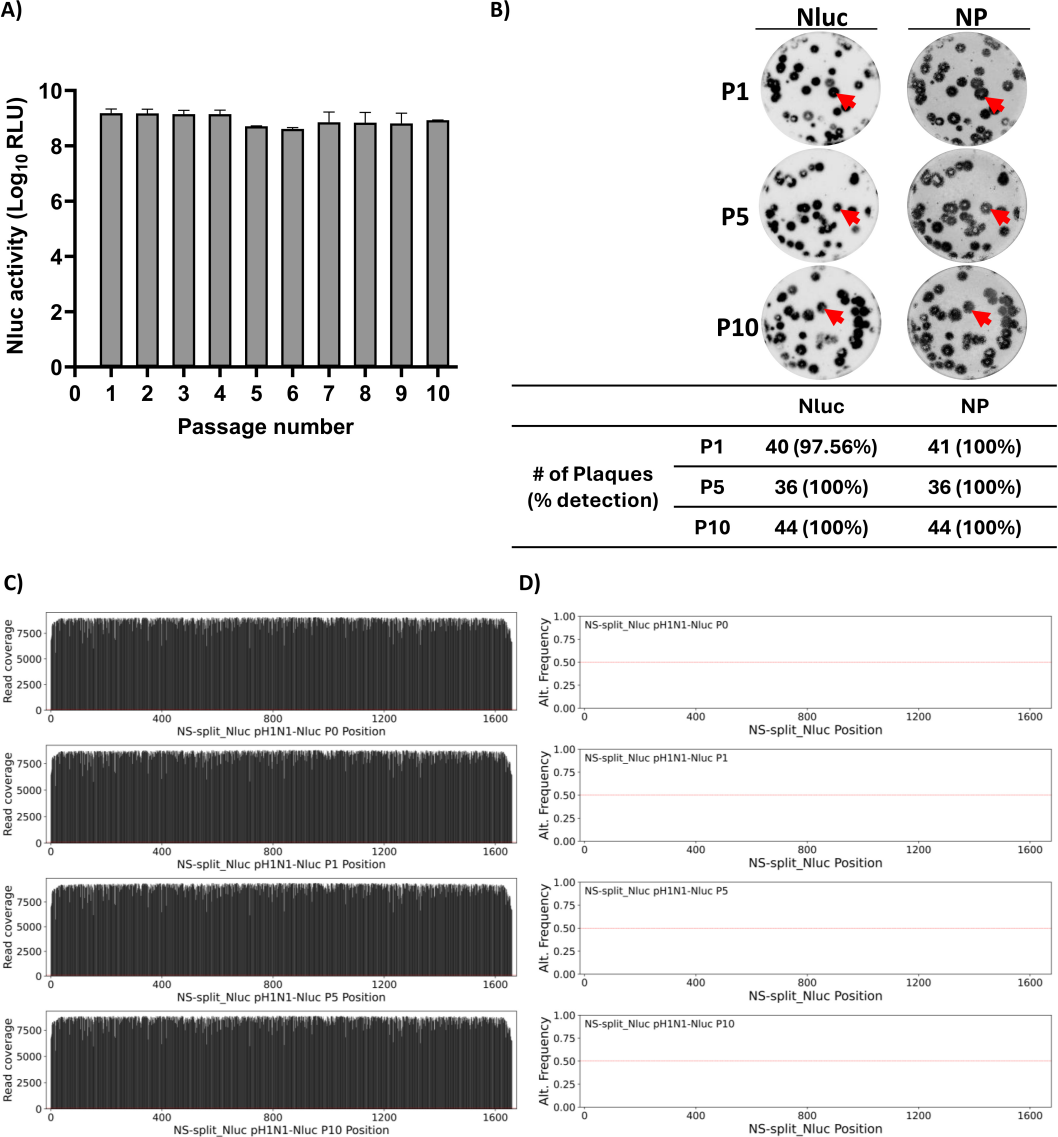
Stability of pH1N1-Nluc virus. MDCK cells were successively infected with pH1N1-Nluc for 10 serial passages. (**A**) Nluc expression in culture supernatants was measured for 10 viral passages. (**B**) Plaques of pH1N1-Nluc viruses, collected at passages 1 (P1), 5 (P5), and 10 (P10), were stained with Nluc substrate (left) or immunostained with a MAb (HB-65) against the viral NP (right). (**C**) Next-generation sequencing (NGS) results covering the NSs-Nluc segment across passages 1, 5, and 10. (**D**) Non-reference allele frequency—The passaged samples were compared to the sequence of the used stock of recombinant pH1N1-Nluc virus (P0) to identify variants. The red line represents 50% allele frequency and would coincide with consensus sequence changes in the viral population.

One important aspect for the use of reporter-expressing recombinant viruses is their genetic and phenotypic stability. Therefore, we next assessed the genetic and phenotypic stability of pH1N1-Nluc. To that end, pH1N1-Nluc was serially passaged in MDCK cells for 10 successive passages (Fig. 10). Nluc expression was analyzed for passages P1–P10, showing stable Nluc expression over the 10 passages (Fig. 10A). By plaque assay, pH1N1-Nluc virus collected at passage 1 (P1), passage 5 (P5), and passage 10 (P10) was positive for Nluc with Nluc expression reaching 97,56% (P1), 100% (P5), and 100% (P10) (Fig. 10B). Importantly, NGS of pH1N1-Nluc collected from P1, P5, and P10 detected Nluc through the 10 serial passages (Fig. 10C) with significant coverage (Table S1). No significant single-nucleotide polymorphisms (SNPs) were detected in the NSs-Nluc viral segment (Fig. 10D), and only a few point mutations were observed in other viral segments (Fig. S2). Contrary to previously described fluorescent-expressing recombinant IAV (10, 20, 24), this finding suggests that pH1N1-Nluc is genotypically and phenotypically stable.

### Generation and characterization of recombinant PR8-Nluc

**Fig 11:**
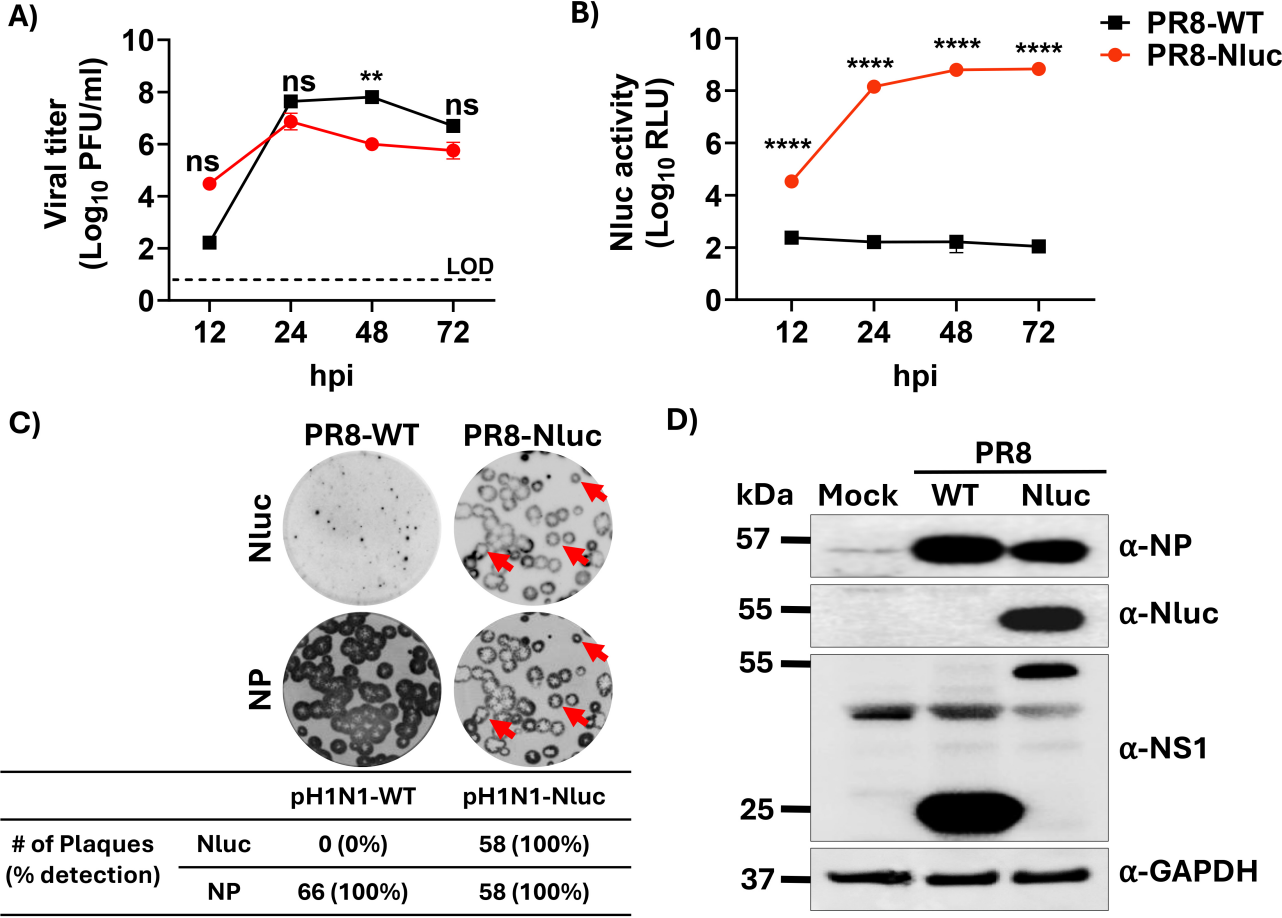
Generation and *in vitro* characterization of PR8-Nluc. (**A**) Replication kinetics of PR8-WT and PR8-Nluc viruses in MDCK cells. Viral titers from culture supernatants of PR8- and PR8-Nluc-infected (MOI 0.01) MDCK cells were determined using immunofocus assay at 12, 24, 48, and 72 hpi. The dotted line indicates the limit of detection (LOD) of the assay. (**B**) Nluc expression in culture supernatants at the indicated time points in panel (**A**). Cell culture supernatants from the viral growth kinetics were used to measure Nluc activity. (**C**) Plaque phenotype of PR8-WT and PR8-Nluc in MDCK cells. Viral plaques were evaluated at 72 hpi. Nluc staining (top) and NP immunostaining (bottom). Red arrows show the co-localization of Nluc staining (top) and viral plaques (bottom). (**D**) Western blots. MDCK cells were infected (MOI = 3) with PR8-WT or PR8-Nluc, or mock-infected. At 12 hpi, cell extracts were prepared, and a Western blot was performed to assess levels of NP, NS1, and Nluc expression. Cellular glyceraldehyde-3-phosphate dehydrogenase (GAPDH) was used as a loading control. Data are represented as mean ± SD. A two-way repeated measures ANOVA with Geisser-Greenhouse correction was used. Post-hoc multiple comparisons were performed using Šídák to compare groups within each time point. The significant differences are indicated (ns = non-significant, **P  <  0.01, ****P  <  0.0001).

To validate the feasibility of incorporating Nluc into the NS segment of a commonly used influenza A/Puerto Rico/8/1934 (H1N1) strain (PR8-WT), we genetically engineered and successfully rescued a recombinant PR8 virus-expressing Nluc (PR8-Nluc) using similar experimental approaches to those described for pH1N1. The replication kinetics of PR8-Nluc were evaluated by plaque assay to assess viral growth dynamics and were compared to those of the parental PR8 (PR8-WT) (Fig. 11). PR8-Nluc and PR8-WT showed comparable growth kinetics at 12, 24, 48, and 72 hpi (Fig. 11A). The presence of Nluc in the same cell culture supernatants reflected a time-dependent increase and plateaued when viral titers remained stable (Fig. 11B). As expected, Nluc activity in the cell culture supernatants of PR8-WT-infected MDCK cells was comparable to background levels (Fig. 11B). In parallel, we also evaluated and compared the plaque morphology of PR8-Nluc to PR8-WT (Fig. 11C). Plaque visualization using the Nluc substrate and immunostaining with the anti-NP HB-65 MAb revealed a comparable number (98.11%) and size of plaques for PR8-Nluc, demonstrating robust and consistent Nluc expression without impairing viral replication (Fig. 11C). Additionally, Nluc expression was confirmed by Western blot using lysates of pH1N1-WT and pH1N1-Nluc-infected MDCK cells (Fig. 11D). Importantly, levels of expression of viral NP were comparable in MDCK cells infected with pH1N1-Nluc or pH1N1-WT (Fig. 1F). Although NS1 protein levels detected by Western blot were not apparently equivalent, the subcellular distribution of NS1 was similar in MDCK cells infected with PR8-WT or PR8-Nluc at 8 hpi (Fig. S1B). Similar to pH1N1-WT and pH1N1-Nluc, both PR8-WT and PR8-Nluc retained the ability to suppress IFNβ activation (Fig. S3), indicating that fusion of Nluc to the C-terminal of PR8 NS1 did not impair its ability to inhibit IFNβ activation. These combined results support the feasibility of implementing the same approach for the generation of other H1N1 viruses for their use in *in vitro* and potentially *in vivo* studies to assess viral replication and to easily track viral infections.

## DISCUSSION

Reporter-expressing IAVs represent an excellent option for assessing viral replication, virulence, and transmission (8, 9, 12, 39, 42, 43). In addition, recombinant IAV-expressing fluorescent and/or luciferase reporter genes can be used to easily identify and/or characterize neutralizing MAbs, antiviral compounds, as well as to evaluate vaccine efficacy (3, 13). Fluorescent reporters tend to be unstable after multiple passages in cell culture and, in several instances, result in attenuation of the virus *in vitro* and *in vivo* (9, 21). Additionally, fluorescent proteins are not optimal for *in vivo* imaging of living animals using IVIS and can only be used to detect the presence of the virus in infected tissues *ex vivo* (9). In contrast, luciferase (e.g., Nluc) reporter genes offer several advantages over fluorescent proteins, including their small size, ATP-independent activity, high stability, and strong signal intensity, making them a more appropriate choice for imaging living animals using IVIS (12, 44, 45).

The NS segment of IAV, which encodes the NS1 protein and, via an alternative splicing mechanism, the NEP, has been frequently used for expressing reporter genes as fusions to the C-terminal domain of NS1 (9, 46). However, these studies have been limited to the expression of fluorescent proteins that often resulted in viral attenuation *in vitro* and *in vivo*. While previous studies have demonstrated the feasibility of generating recombinant IAV-expressing Nluc from different viral segments (e.g., PB2, PB1, PA, HA, NS) (15–19), the feasibility of generating and detecting Nluc expression from recombinant seasonal H1N1 IAV from the NS segment has not yet been explored. Herein, we cloned Nluc fused to the C-terminal end of NS1 into a modified NS segment where NS1 and NEP are expressed from a single transcript. Using plasmid-based reverse genetics, we successfully generated a recombinant pH1N1-Nluc virus. *In vitro,* pH1N1-Nluc has comparable levels of viral replication and a similar plaque phenotype to pH1N1-WT. The advantages of pH1N1-Nluc-expressing Nluc include ease of identifying the presence of the virus in infected cells, characterization of neutralizing MAbs or antivirals. Thus, pH1N1-Nluc can be used to interrogate large libraries of biologics to identify those with neutralizing or antiviral activity for their potential use as therapeutics for viral infections. Our results are consistent with previous studies using reporter viruses for the identification of therapeutics against IAV (3, 14, 20, 47, 48).

Our *in vivo* experiments in B6 mice suggest that pH1N1-Nluc virus has similar virulence and similar levels of viral replication in NT and lungs as pH1N1-WT virus. Importantly, Nluc activity in mice infected with pH1N1-Nluc was dose-dependent, with higher levels of Nluc activity in B6 mice infected with a higher number of infectious viruses. Levels of Nluc expression correlated with viral titers, providing an additional method to detect pH1N1 infection and to quantify the presence of the virus in infected mice. Importantly, expression of Nluc did not affect virus replication or pathogenicity in mice, contrary to viruses expressing fluorescent proteins (3, 10, 20, 21). Our initial studies in mice were further confirmed in ferrets, where we were able to detect viral infections in both infected and direct contact naïve animals at levels comparable to pH1N1-WT but with the advantage of being able to detect Nluc luminescence as a surrogate for viral infection. Our stability studies suggest that pH1N1-Nluc is more genetically and phenotypically stable than recombinant IAV-expressing fluorescent proteins (10, 20, 24). The feasibility of implementing this approach to generate other IAV-expressing Nluc from the NS segment was further demonstrated with the generation of a recombinant PR8-Nluc that retained similar replication efficiency and plaque phenotype as compared to PR8-WT in MDCK cells.

In summary, we describe the generation of a replication-competent recombinant pH1N1 virus-expressing Nluc from a modified NS segment that is genetically and phenotypically stable and biologically comparable to pH1N1-WT virus both *in vitro* and *in vivo* for real-time tracking of viral infection in living mice using IVIS. This Nluc-expressing virus can serve as a robust platform for the identification of biologics with antiviral activity using HTS approaches. Moreover, we have also developed a similar Nluc-expressing PR8 virus, one of the most used and validated IAVs in influenza research.

## MATERIALS AND METHODS

### Cells and viruses

MDCK and human 293T cells were obtained from ATCC and grown in Dulbecco’s modified Eagle’s medium (DMEM: Gibco) supplemented with 5% fetal bovine serum (FBS) and 1% PSG (penicillin, 100 units/mL: streptomycin 100 UG/mL; L-Glutamine, 2 mM) at 37°C with 5% CO_2_. The pH1N1-WT, pH1N1-Nluc, PR8-WT, and PR8-Nluc were propagated in MDCK cells as previously described (49).

### Rescue of recombinant pH1N1-Nluc and PR8-Nluc viruses

Ambisense pDZ plasmids were used for the rescue of pH1N1-WT, pH1N1-Nluc, PR8-WT, and PR8-Nluc (50–52). Co-cultures (10^6^ cells/well) of 293T and MDCK cells (1:1) were co-transfected in suspension on six-well plates with eight pH1N1 or PR8 ambisense pDZ plasmids (pDZ-PB2, -PB1, -PA, -HA, -NP, -NA, -M, -NS, or -NS-Nluc). At 24 h after plasmid transfection, the media was removed, and the cells were incubated in DMEM with 0.3% bovine serum albumin (BSA) and 1% PSG containing 0.5 µg/mL tosylsulfonyl phenylalanyl chloromethyl ketone (TPCK)-treated trypsin (Sigma). At 48 h post-transfection, media was collected and used to infect fresh monolayers of MDCK cells in six-well plates (10^6^ cells/well). At 48 h post-infection, rescued viruses were confirmed by standardized HA assay (53) using turkey red blood cells (RBCs) (Lampire Biological Laboratories, USA). Recovered viruses were plaque purified, and viral stocks were propagated in MDCK cells at 37°C in a 5% CO_2_ incubator for 3–4 days. Cell culture supernatants were collected, centrifuged at 2,000 rpm for 5′ at room temperature, aliquoted, and stored at −80°C. For infections, virus stocks were diluted in phosphate-buffered saline (PBS) 0.3% BSA and 1% PSG. Following infection, cell monolayers were maintained in DMEM with 0.3% BSA and 1% PSG containing 0.5 µg/mL TPCK-treated trypsin. Viral titers (PFU/mL) were calculated by standard plaque assay in MDCK cells as previously described (9, 42).

### Western blots

Cell extracts from mock or virus-infected (MOI = 3) MDCK cells were collected and lysed in RIPA buffer. Proteins from lysates were separated by 12% SDS-PAGE, transferred to a nitrocellulose membrane, blocked in 5% fat-free dried milk in PBS containing 0.1% Tween 20 (PBS-T), and incubated overnight at 4°C with specific primary MAb or polyclonal antibody (PAb) against NS1 (1:1,000; PAb 1-73SW) (54), NP (1:1,000; MAb HB-65, Kerafast), or Nluc (1:1,000; Anti-NanoLuc MAb, Promega). Anti-glyceraldehyde 3-phosphate dehydrogenase MAb (1:5,000; Abcam, ab9484) was used as an internal loading control. Bound primary antibodies were detected by HRP (horseradish peroxidase)-conjugated secondary antibodies (1:2,000 dilution). Proteins were detected by chemiluminescence using a Molecular Imager Chemi Doc-XRS (Bio-Rad) (26).

### Virus growth kinetics

Multicycle growth kinetics were conducted in MDCK cells (12-well plate format, 5 × 10^5^ cells/well, triplicates). Briefly, subconfluent monolayers of MDCK cells were infected (MOI of 0.01) with the indicated viruses. After 1 h of viral adsorption, cell monolayers were overlayed with DMEM including 0.3% BSA, 1% PSG, and 0.5 µg/mL TPCK-treated trypsin and incubated at 37°C. At 24, 48, 72, and 96 hpi, viral titers in cell culture supernatants were determined by immunofocus assay (fluorescent-forming units/mL) using the NP MAb HB-65 as previously described (9). Nluc expression in the cell culture supernatants was quantified using a Nano-Glo luciferase substrate (Promega).

### Plaque assays and immunostainings

Subconfluent monolayers of MDCK cells (six-well plate format, 10^6^ cells per well) were infected with the indicated viruses for 1 h at room temperature. After viral adsorption, the virus inoculum was removed, and the cell monolayers were overlayed with infection media (DMEM 0.3% BSA, 1% PSG, 0.5 µg/mL TPCK-treated trypsin) containing agar. The plates were incubated at 37°C for 72 h. Then, plates were fixed overnight in 4% paraformaldehyde (PFA), and the overlay was removed. To visualize Nluc expression, plates were treated with Nano-Glo luciferase substrate and imaged under a Chemidoc (Bio-Rad). After detection of Nluc expression, plates were permeabilized with 0.5% Triton X-100 in PBS for 15 min and immunostained using the NP MAb HB-65. Immunostaining was developed using the vector kits (Vectastain ABC kit for mouse and DAB HRP substrate kit; Vector) following the manufacturer’s specifications.

### Cell-based IFN bioassay

To assess IFNβ promoter activation *in vitro*, MDCK pIFNβ-GFP/IFNβ-FFluc cells expressing GFP and FFluc under the control of the IFNβ promoter (29, 55) were cultured in 12-well plates (5 × 10^5^ cells/well, triplicates) and mock-infected or infected (MOI = 1) with pH1N1-WT, pH1N1-Nluc, PR8-WT, or PR8-Nluc and incubated for 12 h. NS1-deficient (ΔNS1) pH1N1 and PR8 viruses were used as positive controls due to their impaired ability to control IFNβ promoter activation (55). IFNβ promoter activation was assessed by GFP expression using a fluorescent microscope and by FFluc activity from cell lysates using Promega Luciferase Assay System and GloMax Microplate Reader (Promega, USA). For pH1N1-Nluc and PR8-Nluc, Nluc activity in cell culture supernatants was quantified using the Promega Nluc assay kit and GloMax microplate reader (Promega, USA). Viral infections were confirmed by immunostaining the cells with a MAb (HT103) against the viral NP (56) and an Alexa Fluor 594 AffiniPure goat anti-mouse IgG (H + L) secondary antibody.

### Immunofluorescence assay to assess NS1 subcellular localization

To assess the localization of NS1, MDCK cells were cultured in six-well plates (1 × 10^6^ cell/well). 24 h later, cells were infected (MOI = 1) with PR8-WT, PR8-Nluc, pH1N1-WT, or pH1N1-Nluc. Mock-infected cells were included as a control. At 8 hpi, the media were discarded, the cells were fixed with 4% PFA for 1 h, and then permeabilized for 10 min with 0.5% Triton X-100. Fixed/permeabilized cells were washed with PBS and incubated with a rabbit PAb anti-NS1 antibody (54) (1:1,000, 1% BSA in PBS) followed by 1 h incubation with goat anti-Rabbit IgG (H + L) cross-adsorbed secondary antibody, Alexa Fluor 488 after washing the cell monolayers with PBS. To ensure comparable infection in both viruses, MDCK monolayers were incubated with a mouse anti-NP HT103 MAb (1:1,000) overnight at 4°C. Cells were then washed with PBS and incubated with Alexa Fluor 594 AffiniPure goat anti-mouse IgG (H + L) secondary antibody for 1 h. After further washes, cells were incubated for 30 minutes with DAPI (4′,6-diamidino-2-phenylindole; 10 mg/mL in PBS 1% BSA). Finally, cells were washed and supplemented with 1 mL of PBS. Fluorescence was visualized using the EVOS M5000 Imaging System (ThermoFisher Scientific, USA).

### Nluc-based microneutralization assay for the identification of neutralizing antibodies

To test the neutralizing activity of MAb KPF1, confluent monolayers of MDCK cells (96-plate format, 5 × 10^4^ cells/well, quadruplicates) were infected with 100 PFU of pH1N1-WT or pH1N1-Nluc for 1 h at 37°C. After viral adsorption, cells were washed and incubated with 100 µL of infection medium containing twofold serial dilutions (starting concentration of 0.25 µg/mL) of KPF1 or PBS, 1% Avicel (Sigma-Aldrich), and 1 µg/mL of TPCK trypsin. The plates were then incubated at 37°C for 24 h. Subsequently, the overlays were removed, and cells were fixed with 4% PFA for 1 h, washed with PBS, and permeabilized with 100 µL of 0.5% Triton X-100 in PBS at room temperature for 15 min. Cells are then incubated with the mouse HB-65 primary antibody against NP for 1 h, followed by staining with an anti-mouse secondary antibody from the Vectastain ABC kit and a DAB peroxidase substrate kit (Vector Laboratories) according to the manufacturer’s recommendations. Viral plaques in each well were quantified using an ImmunoSpot plate reader. The viral titers were calculated as previously described (57–59). The mean ± SD of viral inhibitions is calculated from quadruplicate wells. Non-linear regression curves and IC_50_ values were determined using sigmoidal dose-response curves on GraphPad Prism.

For the Nluc activity-based microneutralization assay, MDCK cells were infected with 100 PFU of pH1N1-Nluc virus for 1 h. Cell monolayers were washed and incubated with 100 µL of infection media containing twofold serial dilutions (starting concentration of 0.25 µg/mL) of KPF1 or PBS, 1 µg/mL of TPCK trypsin, and incubated for 48 h. At 48 hpi, supernatants were collected, and Nluc activity was measured by adding Nluc substrate (1:50) at a 1:1 ratio. A Glowmax plate reader was used to read the Nluc activity in the cell culture supernatants. The mean ± SD of Nluc activity inhibitions was calculated from quadruplicate wells. Non-linear regression curves and IC_50_ values were determined using sigmoidal dose-response curves on GraphPad Prism.

### Nluc-based antiviral assay for the identification of antivirals

To test the antiviral activity of Ribavirin, confluent monolayers of MDCK cells in a 96-well plate (5 × 10^4^ cells/well, quadruplicates) were infected with 100 PFU of pH1N1-WT or pH1N1-Nluc for 1 h at 37°C. After viral adsorption, cells were washed and incubated with 100 µL of infection media containing twofold serial dilutions (starting concentration of 100 µM) of Ribavirin or PBS, 1% Avicel (Sigma-Aldrich), and 1 µg/mL of TPCK trypsin, and then incubated at 37°C for 24 h. Then, the overlay was removed, and cells were fixed with 4% PFA for 1 h. Subsequently, fixed cell monolayers were washed with PBS and permeabilized with 100 µl of 0.5% Triton X-100 in PBS at room temperature for 15 min. Cells were then incubated with the mouse HB-65 primary antibody against the viral NP for 1 h, followed by staining with an anti-mouse secondary antibody from the Vectastain ABC kit and a DAB peroxidase substrate kit (Vector Laboratories), according to the manufacturer’s recommendations. Viral plaques in each well were determined using an ImmunoSpot plate reader. The viral titers were calculated as previously described (57–59). The mean ± SD of viral inhibitions was calculated from quadruplicate wells. Non-linear regression curves and IC_50_ values were determined using sigmoidal dose-response curves on GraphPad Prism.

For the Nluc activity-based antiviral assay, MDCK cells were infected with 100 PFU of pH1N1-Nluc virus for 1 h. Cell monolayers were washed and incubated with 100 µL of infection media containing twofold serial dilutions (starting concentration of 100 µM) of Ribavirin or PBS, 1 µg/mL of TPCK trypsin, and incubated at 37°C in a 5% CO_2_ incubator. At 48 hpi, supernatants were collected, and Nluc activity was measured by adding Nluc substrate (1:50) at a 1:1 ratio. A Glowmax plate reader was used to read Nluc activity in the cell culture supernatants. The mean ± SD of Nluc activity was calculated from quadruplicate wells. Non-linear regression curves and IC_50_ values were determined using sigmoidal dose-response curves on GraphPad Prism.

### Mouse experiments

Six-week-old female C57BL/6J (B6) mice were purchased from the Jackson Laboratory (Maine, USA) and maintained in an animal facility at Texas Biomed under specific pathogen-free conditions. For viral infection, cohorts of mice were anesthetized by gaseous sedation in an isoflurane chamber and intranasally inoculated with 10^2^, 10^3^, 10^4^, or 10^5^ PFU of pH1N1-WT or pH1N1-Nluc in a total volume of 50 µL (*n* = 5 mice/group). A group of mock-infected mice (*n* = 4) was included as a control. To minimize the number of animals, the mock-infected group was the same for the pH1N1-Nluc- and pH1N1-WT-infected groups. The body weight, survival data, and images for all groups were collected at the same time. Morbidity (changes in body weight) and mortality (% survival) of pH1N1-WT or pH1N1-Nluc in infected animals (*n* = 5/group) were determined for 15 days. Mice with 25% wt loss from the initial body weight were determined to reach the experimental endpoint and were humanely euthanized. Survival curves were plotted according to the Kaplan-Meier. Animals in the body weight group were also used to detect Nluc expression using an IVIS Spectrum multispectral imaging system. Briefly, mice were anesthetized on days 1, 2, 4, 6, 8, and 10 post-infection with isoflurane and injected with a 1:10 dilution in PBS of the Nano-Glo luciferase substrate retro-orbitally in a final volume of 100 µL and immediately imaged in the IVIS Spectrum multispectral imaging system. Data acquisition and analysis of Nluc expression in infected mice were conducted using the Aura program (AMI spectrum). Flux measurements were acquired from the region of interest. The scale used is indicated in each of the figures. Another group of mice was similarly infected and humanely euthanized at 2 and 4 dpi (*n* = 3/group/time point). Animals in the mock-infected group were the same for mice infected with pH1N1-Nluc and pH1N1-WT. Before euthanasia, Nluc expression in the entire animals was determined as described above by anesthetizing the animals with isoflurane and retro-orbitally injecting with 100 µL of Nano-Glo luciferase substrate (1:10). After imaging, lungs and NT from naïve and infected mice were collected after euthanasia and homogenized in 1 mL of PBS using a Precellys tissue homogenizer (Bertin instruments) for 20 s at 7,000 rpm. Tissue homogenates were centrifuged at 12,000 × *g* at 4°C for 5 min. Supernatants were collected and titrated by plaque assays and immunostaining as previously described (27, 60).

### Ferret experiments

To evaluate the feasibility of using pH1N1-Nluc to study virus pathogenicity and transmission in ferrets, outbred 4-month-old castrated male Fitch ferrets were obtained from Triple F Farms (Gillett, PA, USA). A contact transmission study was conducted with six ferrets per group: two ferrets were DI (10^6^ PFU/ferret) with either pH1N1-WT or pH1N1-Nluc, and four naive ferrets were assigned as contacts (1:2 ratio). Briefly, on day 0, infected ferrets were anesthetized and inoculated intranasally with 10^6^ PFU of pH1N1-WT or pH1N1-Nluc and housed individually. At 24 hpi, two naive contact ferrets were introduced into each cage of the infected ferrets, resulting in three ferrets per cage (one infected: two contact). Body weights were recorded at 0, 2, 4, 6, and 7 dpi. Nasal washes were collected from anesthetized ferrets on 2, 4, and 6 dpi to determine viral titers. On day 7, ferrets were humanely euthanized under anesthesia via exsanguination followed by intracardiac injection of Sleepaway euthanasia solution (Fort Dodge, Sodium Pentobarbital). NT, OLs, trachea, and lungs were collected for viral quantification by plaque assay and immunostaining as described above (27, 60).

### Genetic and phenotypic stability of pH1N1-Nluc *in vitro*

MDCK cells (six-well plates, 10^6^ cells/well) were plated and infected (MOI of 0.01) with pH1N1-Nluc and incubated at 37°C in a 5% CO_2_ incubator until ~70%–80% cytopathic effect (CPE) was observed. Cell culture supernatants were harvested and diluted (1:100) for subsequent serial passages in fresh MDCK cells (six-well plates, 10^6^ cells/well) for a total of 10 passages (P). At each passage, cell culture supernatants were used to assess Nluc expression and viral titration using the plaque infectivity assay and immunostaining with the NP MAb HB-65 (27). Viral RNA from passages 1 (P1), P5, and P10 was extracted using TRIzol reagent (ThermoFisher Scientific, US), for whole-genome sequencing using the next-generation sequencing platform MinION (Oxford Nanopore Technologies). Sample libraries were prepared using the Native Barcoding Kit 24 V14 (SQK-NBD114.24, Oxford Nanopore Technologies) and ran on R10.4.1. Flow Cells (FLO-MIN114, Oxford Nanopore Technologies) per the manufacturer’s instructions. The read length stats with n50 v1.7.0, and then the raw reads were trimmed using nanoq v0.10.0 (61). Bases with quality PHRED quality scores less than 7 were removed along with the first and last 25 bp of each read. Reads less than 500 bp after trimming were removed from downstream analyses. Filtered reads were mapped to the pH1N1-Nluc reference sequence using minimap2 v2.28-r1209 (62) and the “-x map-ont” option for mapping long error-prone nanopore reads. Mapping rates were calculated with SAMtools flagstat v1.21 (63), and reads with mapping quality (MAPQ) scores less than 40 were removed, as well as reads mapping to less than 1,000 bp of the reference. We estimated coverage across each genome with MosDepth v0.3.10 (64). We reevaluated indel quality scores and called variants using LoFreq v2.1.5 (65). We limited the depth of coverage (--max-depth) for variant calling at 5,000× and removed the default filters applied by LoFreq. We used custom filters to remove any variants that were present in less than 25% of reads, with read depths less than 100×, or were in homopolymer regions of 3 bp or larger.

### Quantifications and statistical analyses

All graphs, calculations, and statistical analyses were performed using GraphPad Prism software version 9.5.1 (GraphPad Software, LLC, USA). Growth kinetics were analyzed using a two-way repeated-measure ANOVA with Geisser-Greenhouse correction. Post hoc comparisons were performed using the Šídák method. Differences in FFluc expression were evaluated using Welch’s one-way ANOVA, followed by multiple comparisons using Dunnett T3 method. Viral replication and titer data were analyzed using mixed-effects ANOVA. Tukey *post hoc* comparisons test was used to compare groups within each timepoint. The significance of differences is indicated as follows: ns = non-significant, *P  <  0.05, ** *P*  < 0.01, *** *P*  <  0.001, ****P < 0.0001.

## Data Availability

All NGS raw data are available under the BioProject PRJNA1288407.

## SUPPLEMENTAL MATERIAL

Supplemental figures and tables spectrum.02150-25-s0001.docx Figures S1 to S3; Table S1.

ASM does not own the copyrights to Supplemental Material that may be linked to, or accessed through, an article. The authors have granted ASM a non-exclusive, world-wide license to publish the Supplemental Material files. Please contact the corresponding author directly for reuse.
