# Supplementary material for: Bioluminescent reporter influenza A viruses to track viral infections
Source: Microbiol Spectr. 2025 Oct 8;13(11):e02150-25. doi: 10.1128/spectrum.02150-25 (PMC12584733; doi:10.1128/spectrum.02150-25)
Supplement: Supplemental figures and tables — Figures S1 to S3; Table S1. [file spectrum.02150-25-s0001.docx]

**Bioluminescent reporter influenza A viruses to track viral infections**

Ramya S. Barre^1,2^, Ahmed Mostafa^1,3^, Kevin Chiem^1^, Rebecca L. Pearl^4,5^, Roy N. Platt^1^, Anastasija Cupic^4,6^, Timothy J. C. Anderson^1^, Ulla G. Knaus^7^, Randy A. Albrecht^4,5^, Adolfo García-Sastre^4,5, 8,9,10,11^, James J. Kobie^12^, Aitor Nogales^13^, Luis Martinez-Sobrido^1,*^

^1^ Texas Biomedical Research Institute, San Antonio, TX, USA.

^2^ Department of Microbiology, Immunology, and Molecular Genetics, University of Texas Health Sciences Center at San Antonio, San Antonio, TX, USA.

^3^ Center of Scientific Excellence for Influenza Viruses, National Research Centre, Giza, Egypt.

^4^ Department of Microbiology, Icahn School of Medicine at Mount Sinai, New York, NY, USA.

^5^ Global Health Emerging Pathogens Institute, Icahn School of Medicine at Mount Sinai, New York, NY, USA.

^6^ Graduate School of Biomedical Sciences, Icahn School of Medicine at Mount Sinai, New York, NY, USA.

^7^ Conway Institute, School of Medicine, University College Dublin, Dublin 4, Ireland

^8^ Department of Medicine, Division of Infectious Diseases, Icahn School of Medicine at Mount Sinai, New York, NY, USA.

^9^ Tisch Cancer Institute, Icahn School of Medicine at Mount Sinai, New York, NY, USA.

^10^ Department of Pathology, Molecular and Cell-Based Medicine, Icahn School of Medicine at Mount Sinai, New York, NY, USA.

^11^ Icahn Genomics Institute, Icahn School of Medicine at Mount Sinai, New York, USA

^12^ Heersink School of Medicine, Infectious Diseases, University of Alabama at Birmingham, Birmingham, Alabama, AL, USA.

^13^ Center for Animal Health Research, CISA-INIA-CSIC, Madrid, Spain.

* Correspondence should be addressed to:

Luis Martinez-Sobrido (lmartinez@txbiomed.org; <https://orcid.org/0000-0001-7084-0804>)

**Supplementary Figure S1. Subcellular localization of NS1 and NS1-Nluc in MDCK cells infected with (A) pH1N1-WT and pH1N1-Nluc, or (B) PR8-WT and PR8-Nluc.** MDCK cells were infected (MOI=1) with pH1N1-WT and pH1N1-Nluc (A) or PR9-WT or PR8N-Nluc (B). Mock-infected cells were included as a control. At 8 hpi, cells were fixed, permeabilized, and stained with anti-NS1 PAb followed by Alexa Fluor 488-conjugated secondary antibody (green). Nuclei were counterstained with DAPI (blue). Viral NP was detected using an anti-NP Mab followed by an Alexa Fluor 594-conjugated secondary antibody (red). Representative fluorescent images of NS1 show comparable cellular and nuclear localization of NS1 and NS1-Nluc. Representative fluorescent images of NP show comparable levels of viral infection. Scale bar: 75 μm.

| Gene | Coverage | SNP(S) |
| --- | --- | --- |
| PB2 | 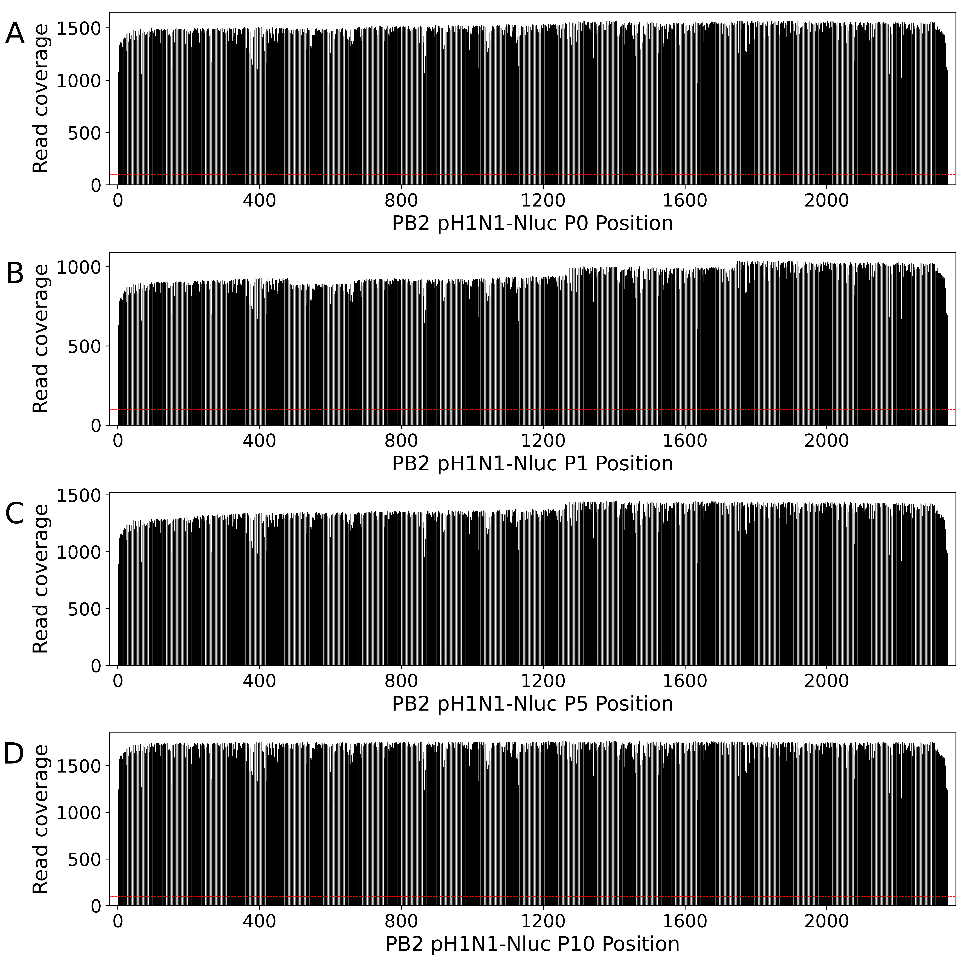 | 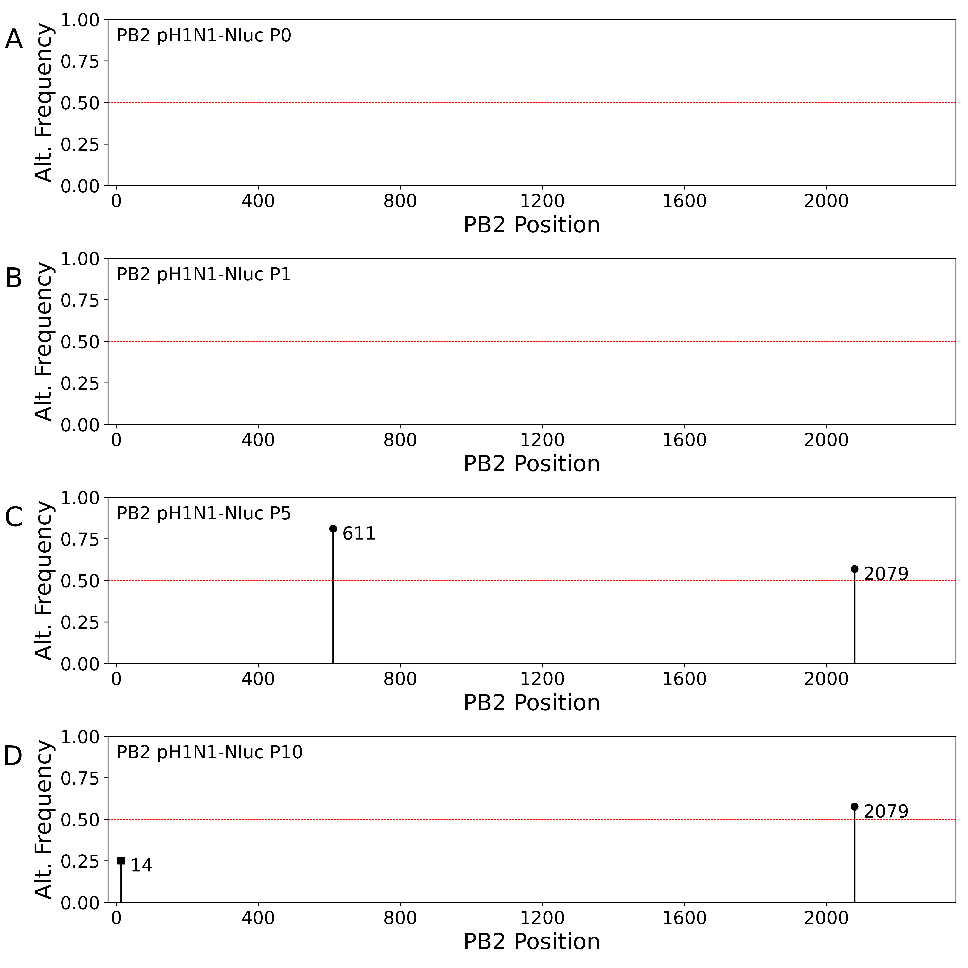 |
| PB1 | 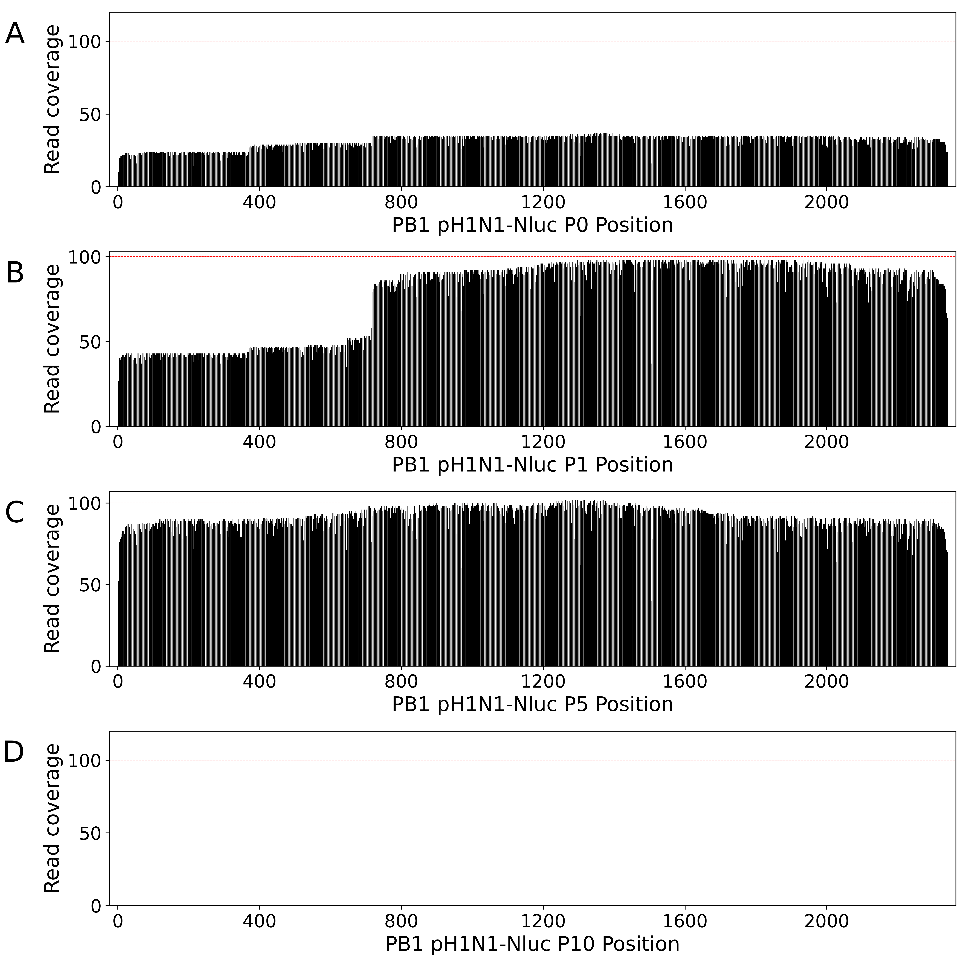 | 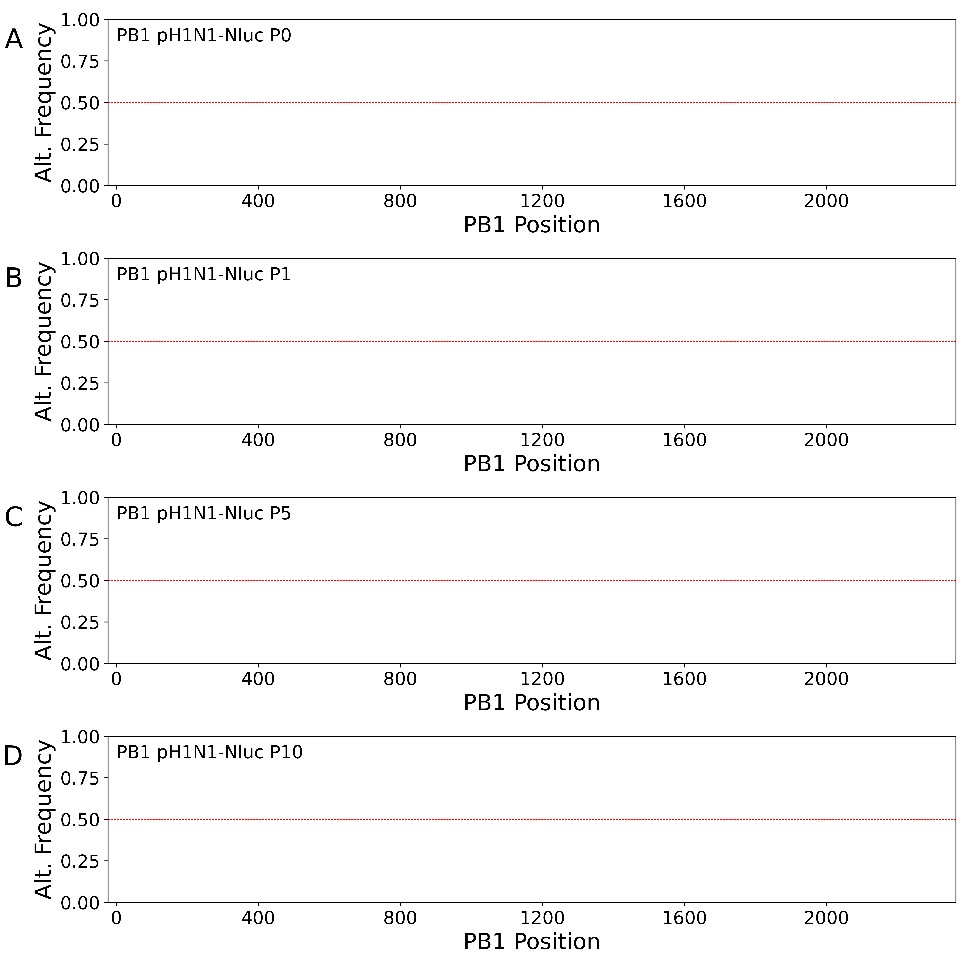 |
| PA | 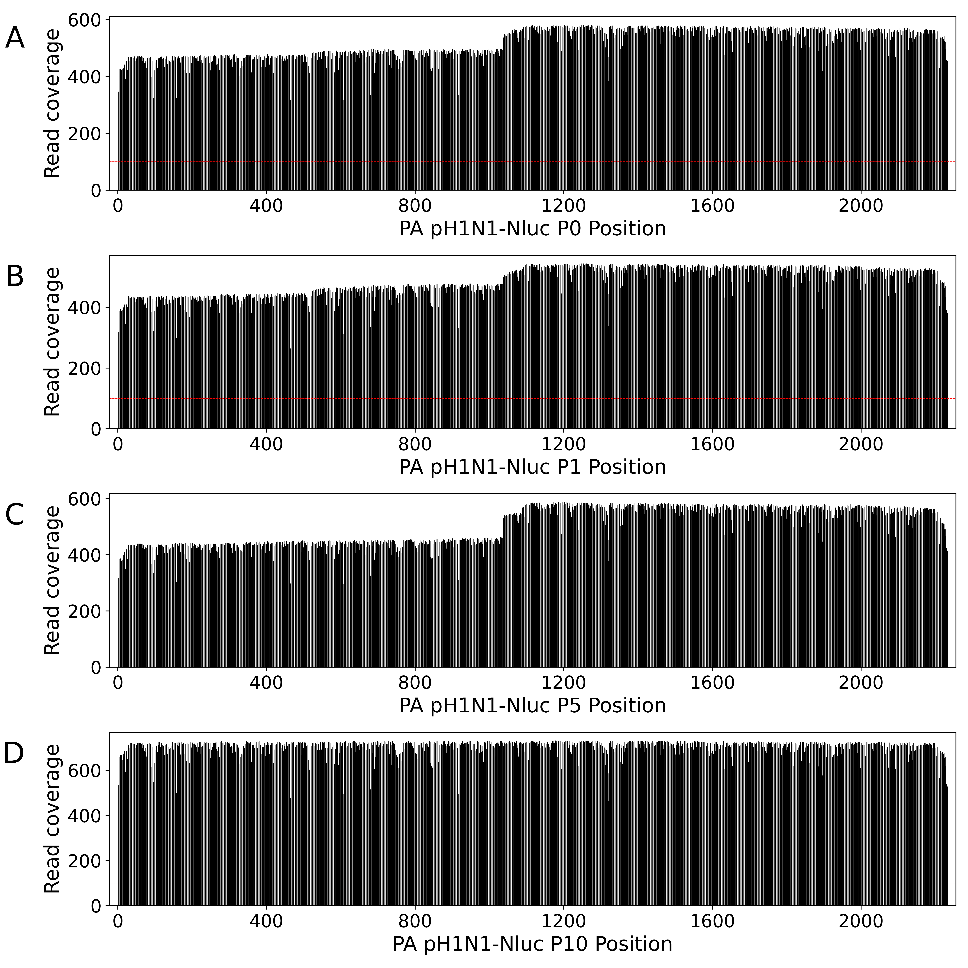 | 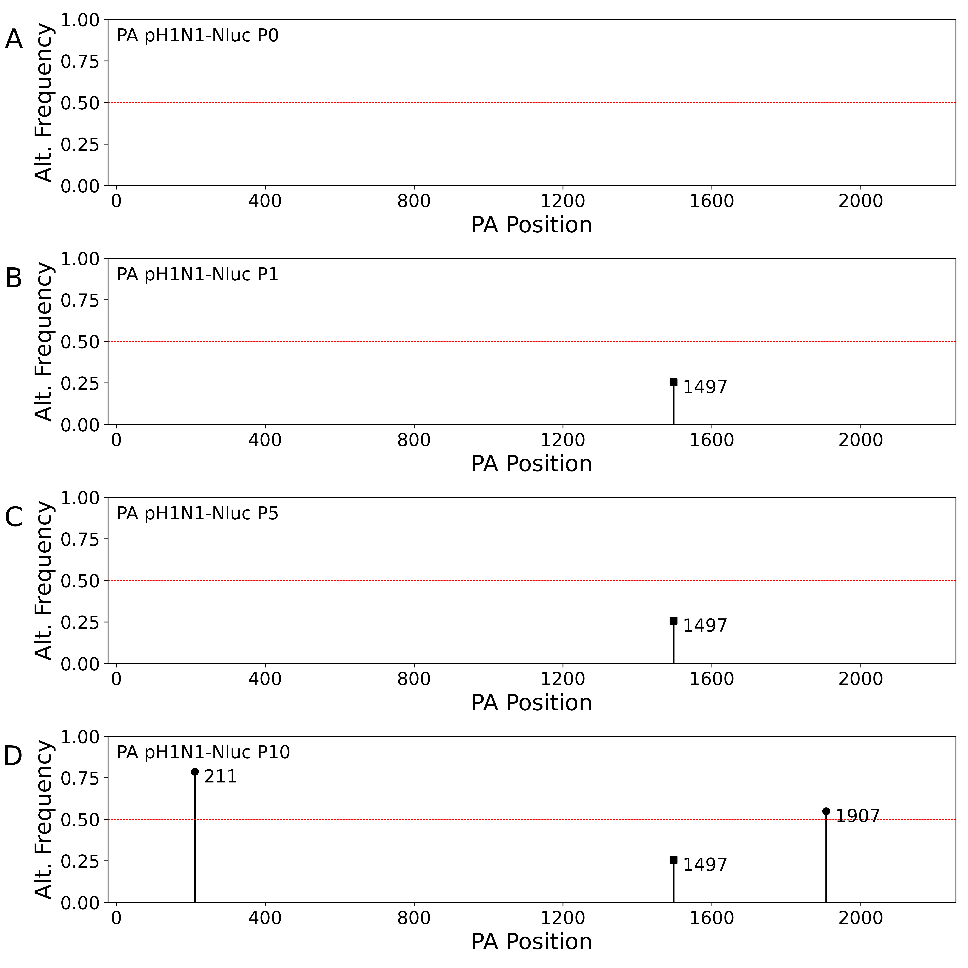 |
| HA | 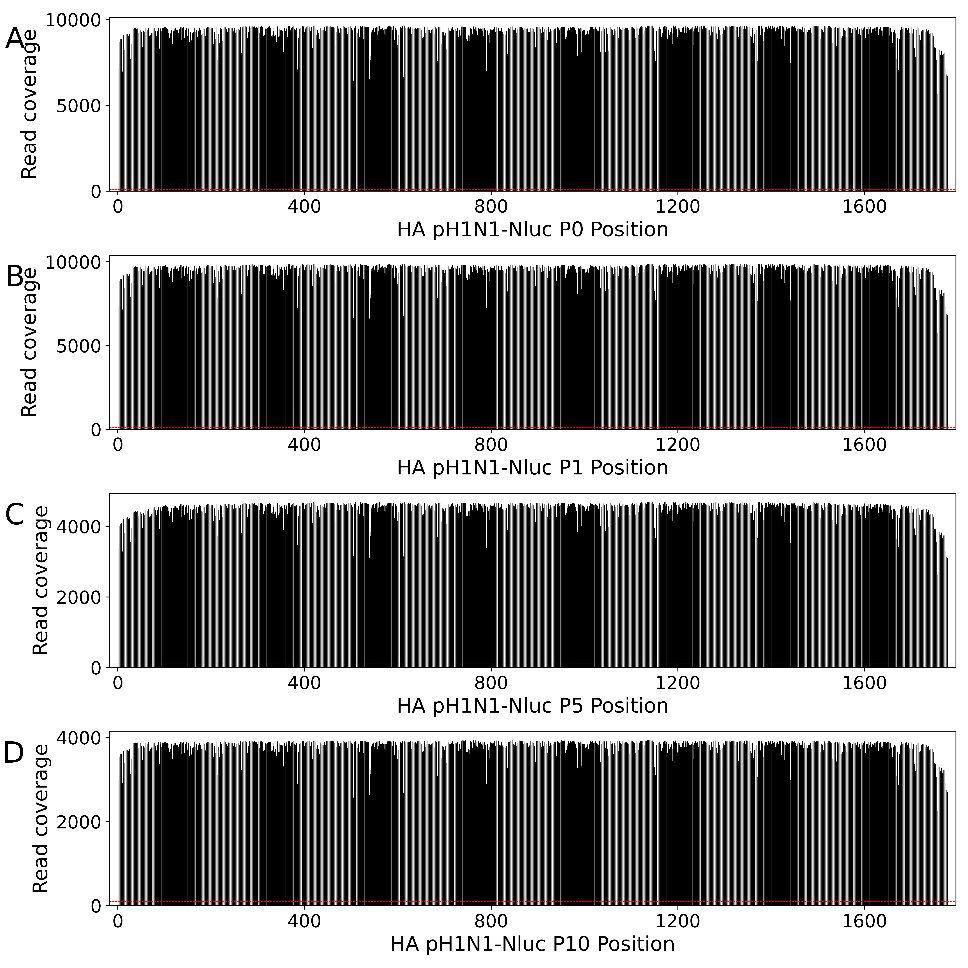 | 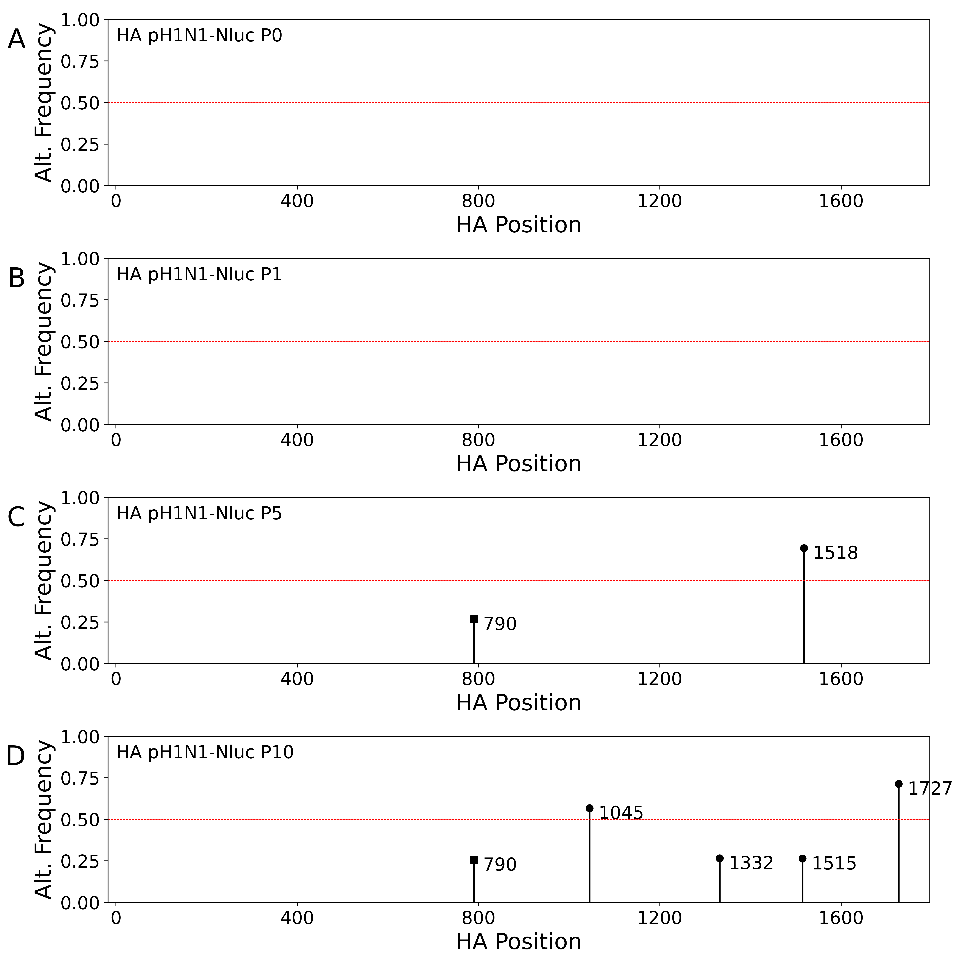 |
| NP | 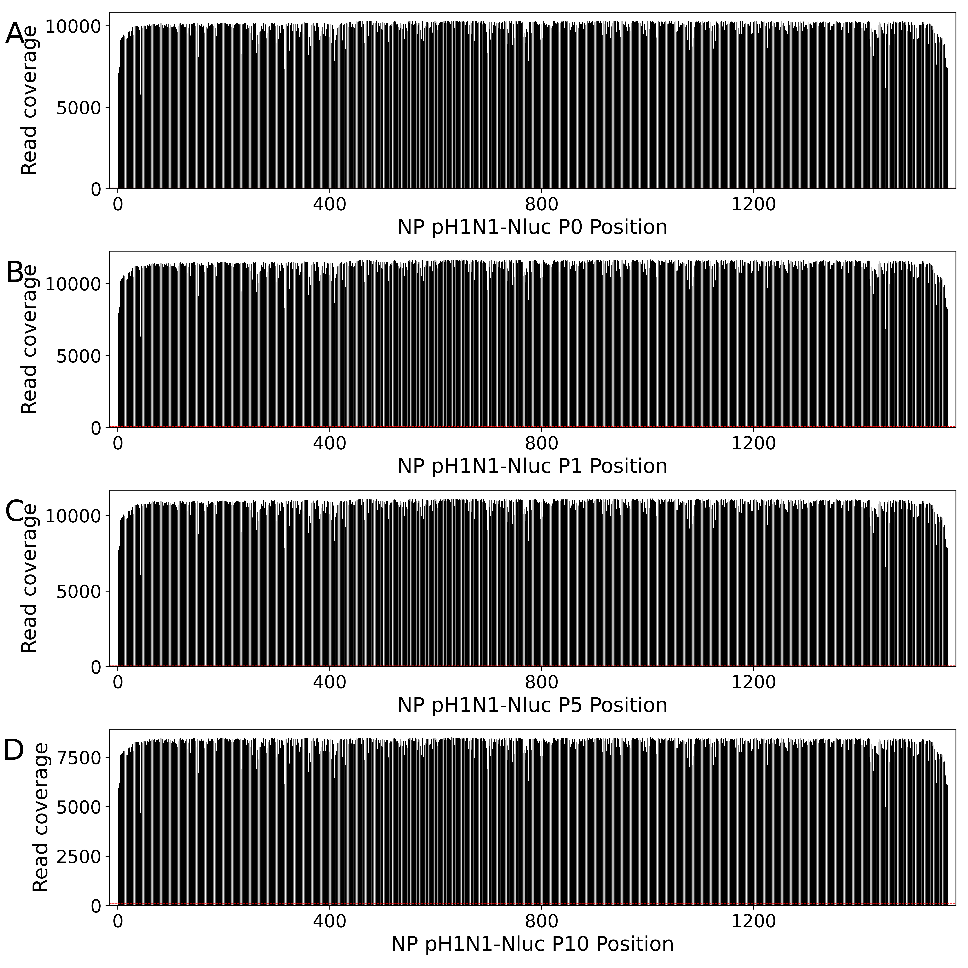 | 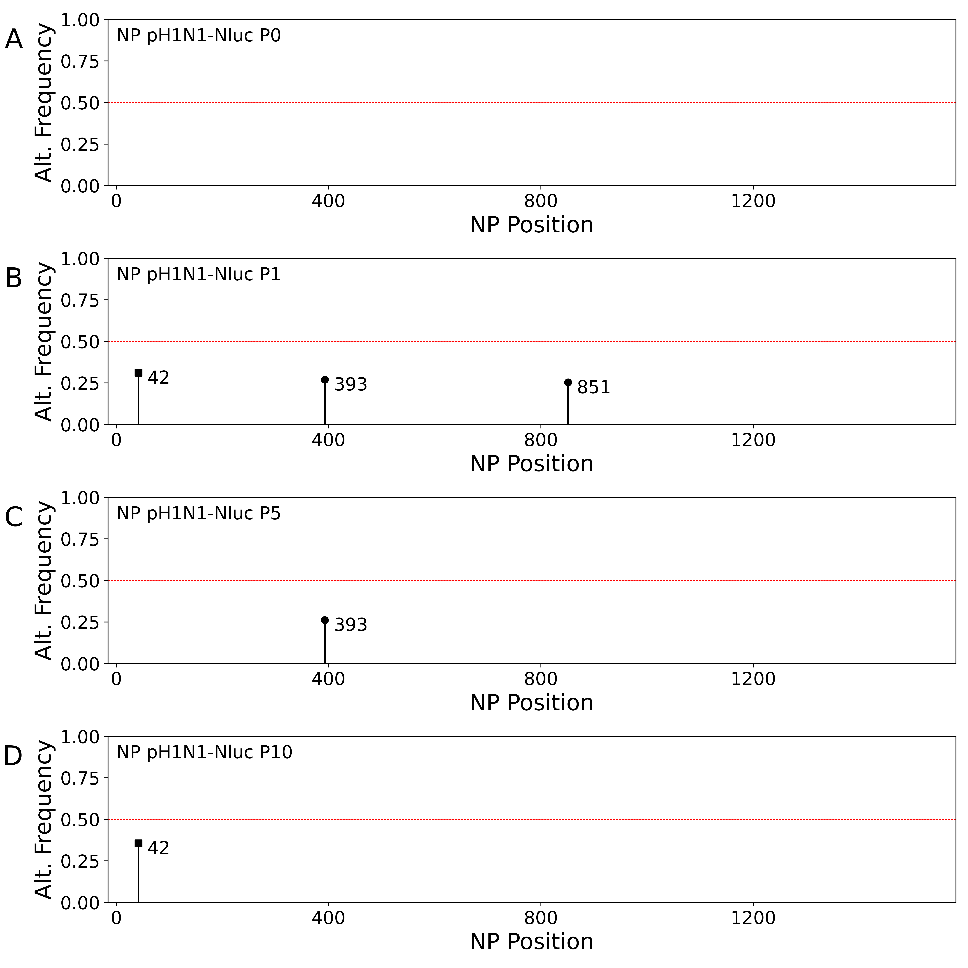 |
| NA | 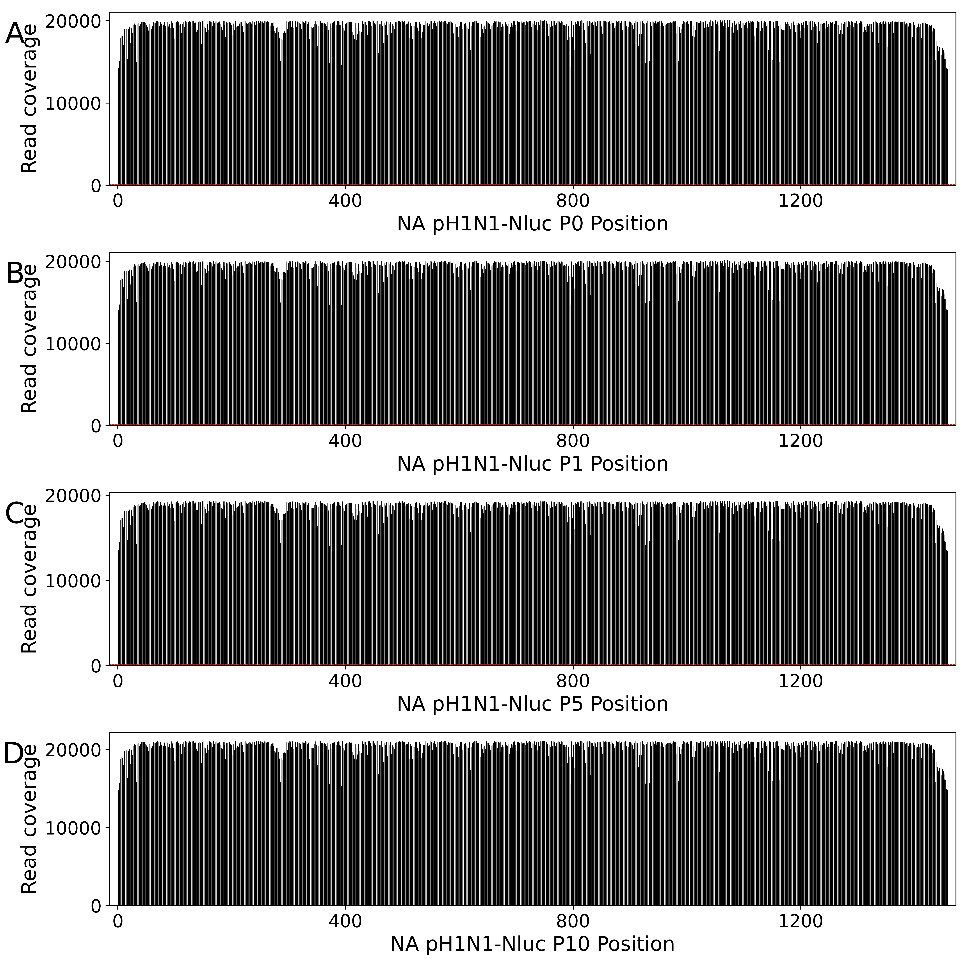 | 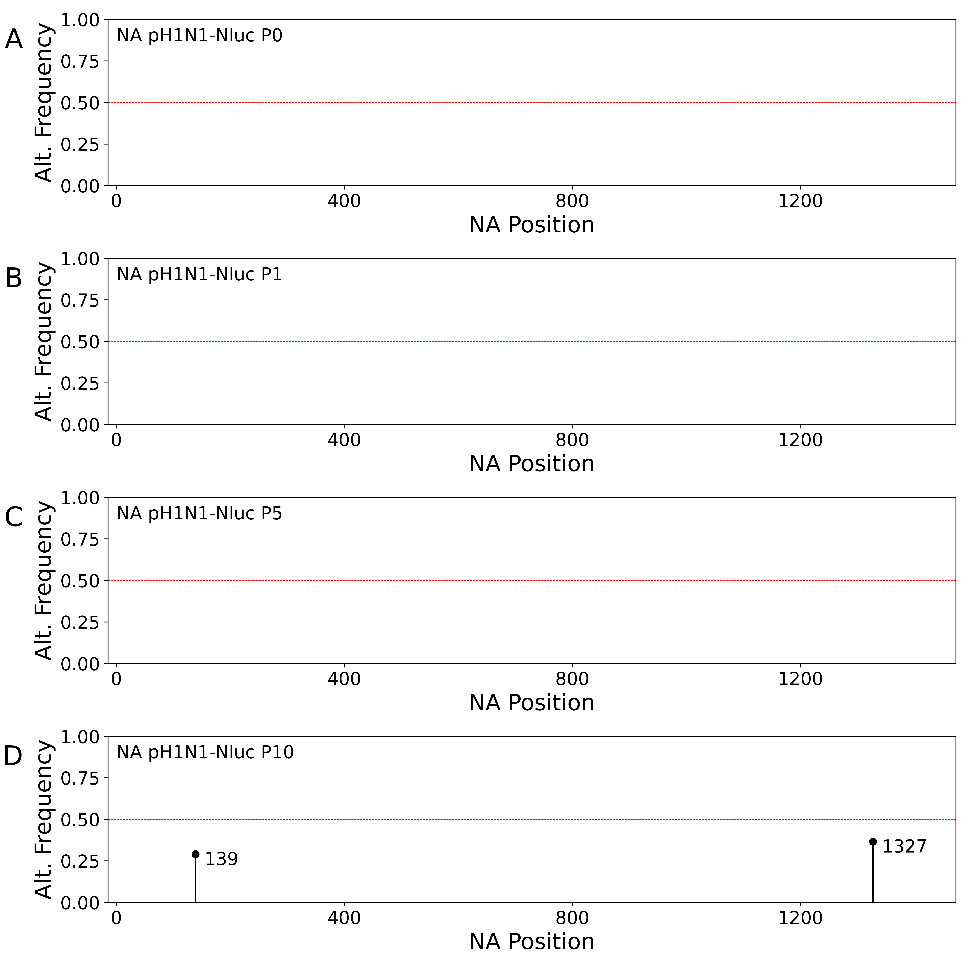 |
| M | 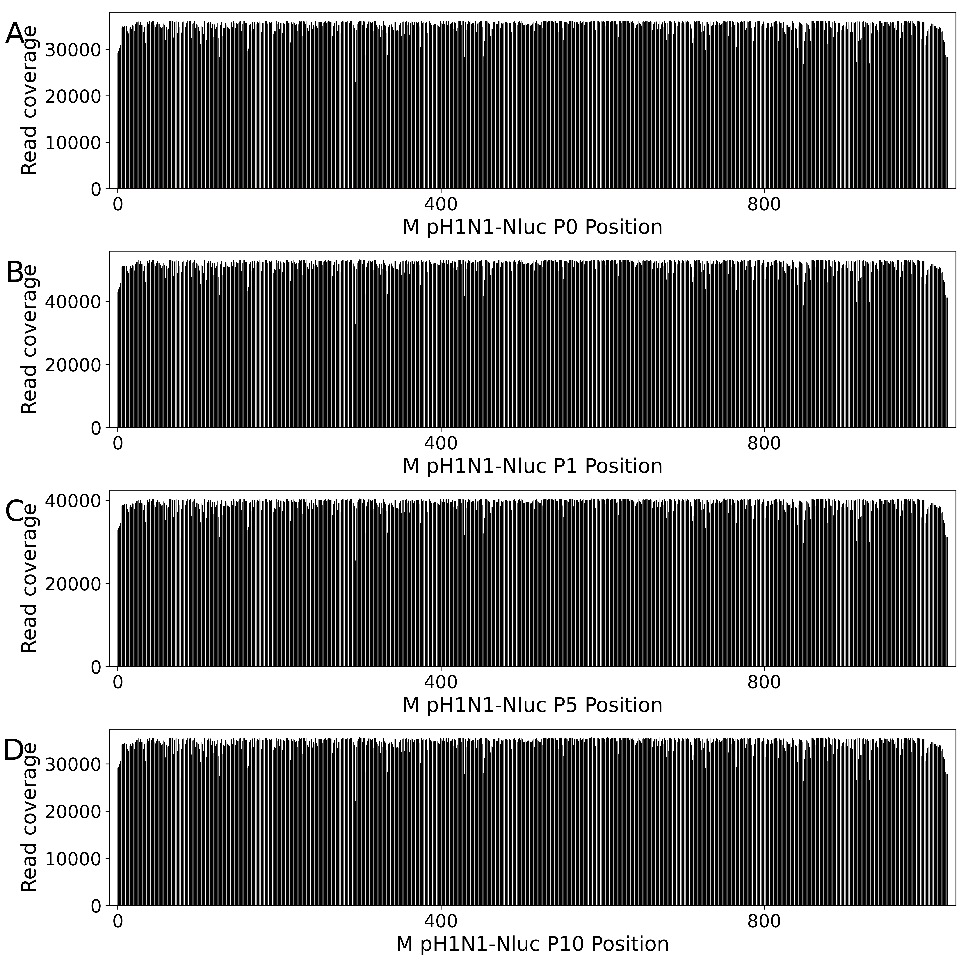 | 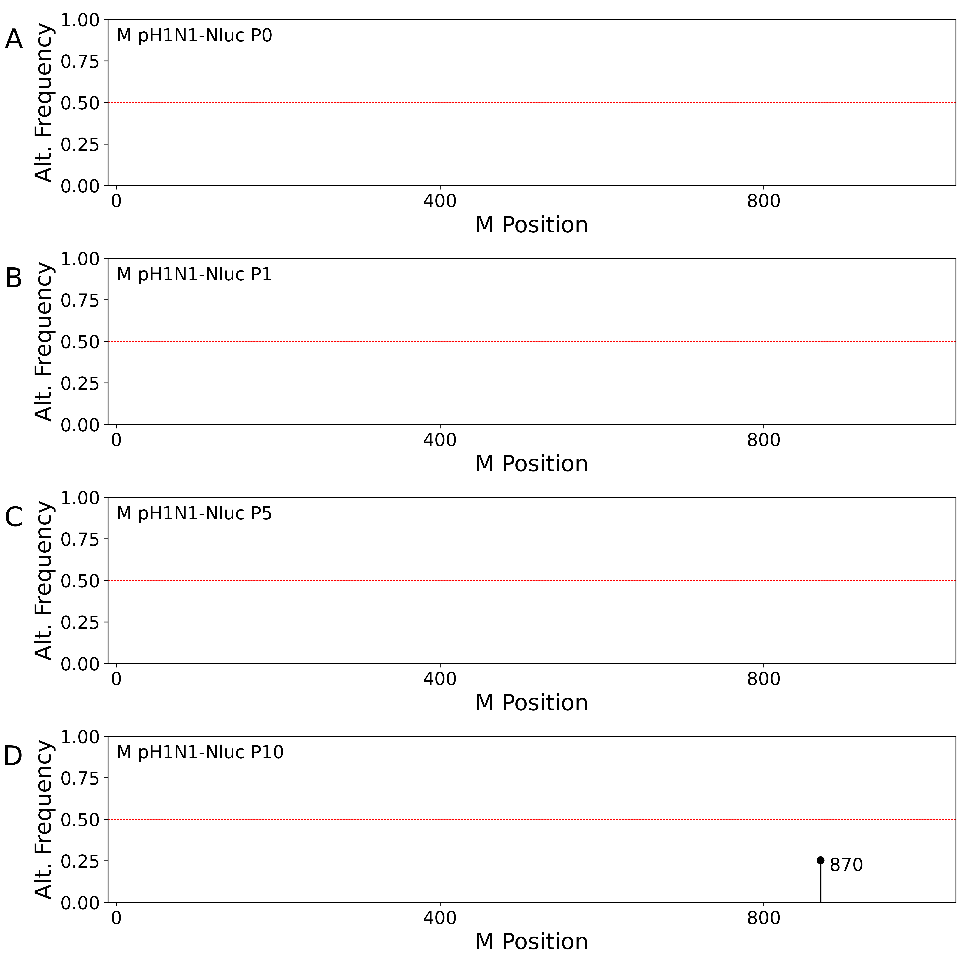 |

**Supplementary Figure S2. Genetic stability of pH1N1-Nluc viral segments PB2, PB1, PA, HA, NP, NA, and M following *in vitro* passaging.** SNPs: Single nucleotide polymorphisms.

**
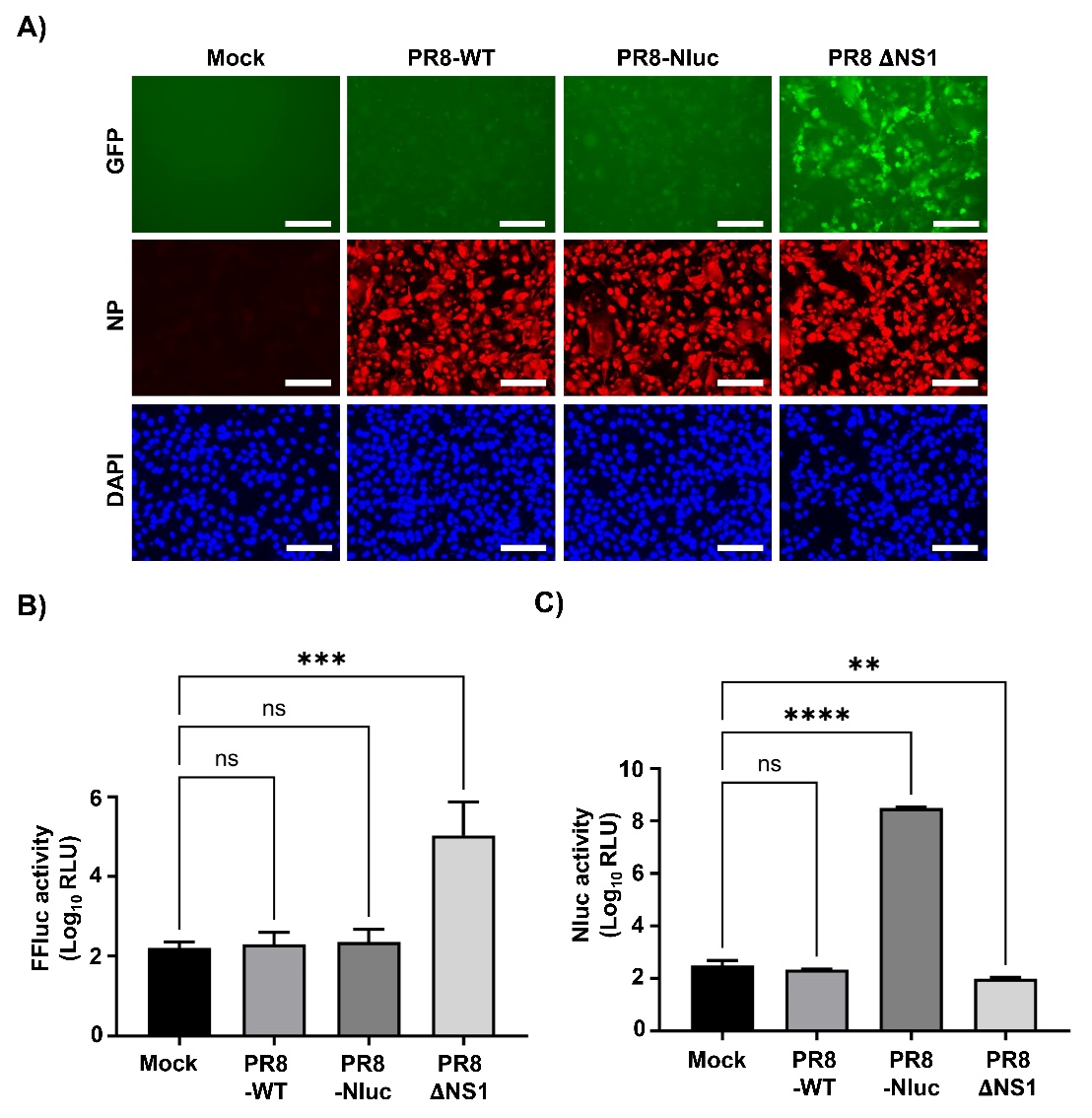
**

**Supplementary Figure S3. PR8-Nluc inhibits IFNβ promoter activation.** MDCK IFNβ-GFP/IFNβ-FFluc cells were mock-infected or infected (MOI=1) with PR8-WT, PR8-Nluc, or PR8 ΔNS1. At 12 hpi, IFNβ promoter activation was analyzed by GFP (**A**) and FFluc (**B**) expression. Nluc expression in the cell culture supernatants from mock- or viral-infected MDCK cells was also determined at the same time after viral infection (**C**). Scale bar: 100 μm. Data are represented as mean +/- SD. A Welch’s one-way ANOVA. Post-hoc multiple comparisons were performed using Dunnett method to compare groups within each time point. The significant differences are indicated (ns=non-significant, **** = *p* < 0.0001, *** = *p* < 0.001, ** = *p* < 0.01).

**Supplementary Table 1. *Data description* -** Sample summary and genome coverage

| Virus_passage | PE reads (1e4) | Alignment Rate | Mean Coverage |
| --- | --- | --- | --- |
| pH1N1-Nluc_P0 | 9 | 99.98% | 8029.3 |
| pH1N1-Nluc_P1 | 131.4 | 99.89% | 9251.6 |
| pH1N1-Nluc_P5 | 145.5 | 99.50% | 7761.8 |
| pH1N1-Nluc_P10 | 116.3 | 99.81% | 8670 |
